# Impact of Integrating Machine Learning in Comparative Effectiveness Research of Oral Anticoagulants in Patients with Atrial Fibrillation

**DOI:** 10.3390/ijerph191912916

**Published:** 2022-10-09

**Authors:** Sola Han, Hae Sun Suh

**Affiliations:** 1 College of Pharmacy, Kyung Hee University, Seoul 02447, Korea; 2 Health Outcomes Division, College of Pharmacy, The University of Texas at Austin, Austin, TX 78712, USA; 3 Department of Regulatory Science, Graduate School, Kyung Hee University, Seoul 02447, Korea

**Keywords:** comparative effectiveness research, propensity score, machine learning, atrial fibrillation

## Abstract

We aimed to compare the ability to balance baseline covariates and explore the impact of residual confounding between conventional and machine learning approaches to derive propensity scores (PS). The Health Insurance Review and Assessment Service database (January 2012–September 2019) was used. Patients with atrial fibrillation (AF) who initiated oral anticoagulants during July 2015–September 2018 were included. The outcome of interest was stroke/systemic embolism. To estimate PS, we used a logistic regression model (i.e., a conventional approach) and a generalized boosted model (GBM) which is a machine learning approach. Both PS matching and inverse probability of treatment weighting were performed. To evaluate balance achievement, standardized differences, *p*-values, and boxplots were used. To explore residual confounding, E-values and negative control outcomes were used. In total, 129,434 patients were identified. Although all baseline covariates were well balanced, the distribution of continuous variables seemed more similar when GBM was applied. E-values ranged between 1.75 and 2.70 and were generally higher in GBM. In the negative control outcome analysis, slightly more nonsignificant hazard ratios were observed in GBM. We showed GBM provided a better ability to balance covariates and had a lower impact of residual confounding, compared with the conventional approach in the empirical example of comparative effectiveness analysis.

## 1. Introduction

The era of big data and machine learning seems to be entering a stage in which it is possible to produce additional high-quality evidence and overcome the common limitations of randomized controlled trials (RCTs), which include short follow-up periods and restricted patients who do not represent real-world patients [1,2]. However, despite its importance, healthcare big data are criticized for their incomplete information on potential unmeasured confounders, which may result in biased estimates of the association between drug exposure and outcomes in comparative effectiveness research [1].

Propensity score (PS) methods are most commonly used to minimize confounding in exposure–outcome associations [3]. However, compared with RCTs, the true PS model remains unknown. Thus, there is a possibility of residual unmeasured confounding or misspecification of the PS model [4].

Recently, machine-learning-based PS methods were introduced to reduce additional confounding [4]. Machine learning methods, such as boosting, have iterative and automated variable selection processes that show good performance in terms of bias reduction or mean squared error when compared with simple logistic regression models [3].

However, studies on machine learning and residual unmeasured confounding are limited. An empirical example of a comparative effectiveness study may provide insights into applying machine learning, and could become an important basis for understanding ways to minimize residual confounding. This could potentially help guide physicians in better healthcare decision making.

This study had two objectives: to compare the ability to balance baseline covariates and to explore and compare the impact of residual unmeasured confounding between the conventional PS and machine-learning-based PS. We used an empirical example, a comparative effectiveness study into patients with atrial fibrillation (AF), a commonly studied topic globally [5,6,7,8,9,10,11], to provide practical insight into the comparative effectiveness research field.

## 2. Materials and Methods

### 2.1. Study Scheme and Data Source

**Figure 1:**
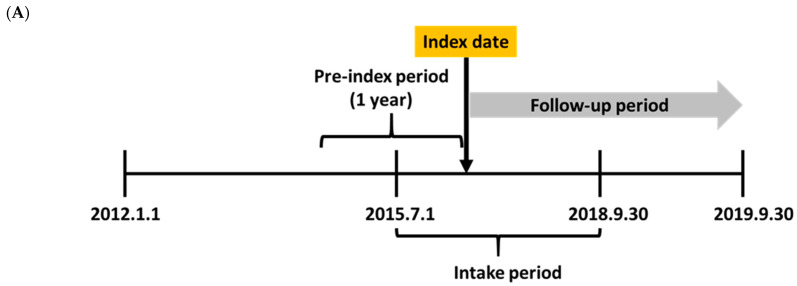

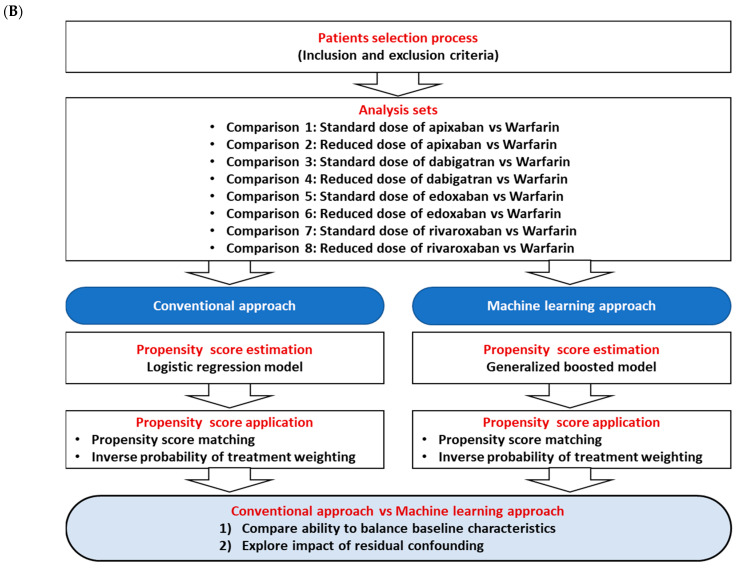
Study scheme. (**A**) Patient selection and follow-up. (**B**) Overall process.

This study utilized an empirical example of a retrospective cohort study to compare the effectiveness of oral anticoagulants (OACs) in patients with AF in Korea. The study scheme is illustrated in Figure 1.

This study used the Health Insurance Review and Assessment Service (HIRA) database (1 January 2012–30 September 2019), which covers the entire Korean population via the country’s universal health insurance system [12]. This database includes the diagnosis, procedure, and medication codes as variables for each medical or pharmacy claim [12]. The diagnoses were coded according to the Korean Standard Classification of Diseases, based on the International Classification of Diseases 10th Revision (ICD-10) codes [12]. 

### 2.2. Study Population and Clinical Outcomes

The study population of interest comprised OAC-naïve patients with AF. Patients who met all the following inclusion criteria were included in this study: initiation of OACs (apixaban, dabigatran, edoxaban, rivaroxaban, or warfarin) between 1 July 2015 and 30 September 2018; ≥1 AF diagnosis code (ICD-10: I48) prior to or on the index date; and age of ≥18 years on the index date.

The following patients were excluded: those in whom OACs (i.e., apixaban, dabigatran, edoxaban, rivaroxaban, or warfarin) were initiated prior to the index date; >1 OAC received at the index date; diagnosis or procedure codes for rheumatic mitral valvular heart disease; diagnosis codes for mitral valve stenosis, venous thromboembolism, end-stage chronic kidney disease, kidney transplant, dialysis, pericarditis, thyrotoxicosis, or hypertrophic cardiomyopathy within one year prior to the index date; both standard and reduced doses for the same index OAC were prescribed at the index date.

In this population, the clinical outcome was stroke or systemic embolism (S/SE). Stroke included ischemic and hemorrhagic stroke. A detailed S/SE definition that was defined based on clinical expert opinion during the follow-up period, is described in Supplementary Table S1. S/SE was defined using a main diagnosis code based on a previous study [13]. 

### 2.3. Study Outcomes

The study outcomes were: (1) the balance of baseline covariates between groups and (2) the impact of residual confounding. We evaluated the balance of baseline covariates between groups using absolute standardized differences, side-by-side boxplots, and a statistical significance test (*p*-value). We evaluated the impact of residual confounding using E-values and negative control outcomes. The E-values for the S/SE were calculated. Pneumonia and urinary tract infections were considered as negative control outcomes. Detailed methods regarding these study outcomes are described in the following sections.

### 2.4. Statistical Analyses—PS Estimation and Balance

We considered the following comparisons: (1) standard dose (10 mg per day) of apixaban vs. warfarin; (2) reduced dose (5 mg per day) of apixaban vs. warfarin; (3) standard dose (300 mg per day) of dabigatran vs. warfarin; (4) reduced dose (220 mg per day) of dabigatran vs. warfarin; (5) standard dose (60 mg per day) of edoxaban vs. warfarin; (6) reduced dose (30 mg per day) of edoxaban vs. warfarin; (7) standard dose (20 mg per day) of rivaroxaban vs. warfarin; and (8) reduced dose (15 mg per day) of rivaroxaban vs. warfarin. Considering the comparability of the results with other studies, the standard- and reduced-dose groups were defined using the recommended dose per day for each non-vitamin K antagonist oral anticoagulants (NOACs) [14]. Reduced doses are recommended if patients have clinical factors (e.g., older age, lower body weight, renal impairment, or concomitant use of a P-glycoprotein inhibitor) that could affect bleeding risk [14]. 

We used logistic regression to estimate the conventional PS, and the generalized boosted model (GBM) to estimate the machine-learning-based PS for each comparison. GBM iteratively generates many simple regression tree models, combining them together to estimate the PS. Similar to logistic regression, GBM models the log odds of the probability of being in the treatment group, g(x) = log(p(x)/(1 − p(x))); however, the g(x) is iteratively updated to find the model with the maximized log-likelihood of g(x) [15,16]. More details on the PS estimation process were published previously [15,16]. For comparison, we used GBM among several machine learning approaches (e.g., LASSO or super learner) because it is the most frequently used in comparative effectiveness studies of patients with AF [6,7,17]. In addition, PS adjustment with GBM reportedly provides better results than other machine-learning approaches [18]. Thus, we believed that using a method that is known to have better performance and is used more frequently could provide more informative results. In addition, it is not necessary to predict the treatment accurately in PS models [19]. It is important to include all confounders in the model. Including variables that accurately predict the treatment but have no bearing on the outcome will cause increased variance in the estimated treatment effect without reducing bias or confounding [19,20]. Therefore, the predictive performance of the PS models cannot be translated into the extent of bias in the treatment effect or the level of balance between the two treatment groups. Thus, we did not evaluate the predictive performance (e.g., confusion matrix, accuracy, precision, or receiver operating characteristics curve). 

To estimate PS, the variables in Supplementary Table S2 were defined using data from one year of the pre-index period. The same variables (covariates) were used for both the logistic regression and the GBM. All variables used for PS estimation were based on RCTs, observational studies, and clinical expert opinion [21,22,23,24,25]. For the logistic regression, a linear relationship was assumed, and no interaction terms were included. For the GBM, the number of trees was 20,000, the interaction depth was 2, the shrinkage parameter was set to 0.01, and the stopping method that selected the optimal number of iterations minimized the standardized differences between the groups [26]. We used these settings because they were introduced as starting points in previously published studies [15,26]. In these studies, it was recommended that the number of tree settings should be large enough to achieve balance, and if balance is not achieved, researchers should consider a more complex model. In this study, the balance was achieved by using these settings and the best balance was found before 20,000 iterations, ranging between 9895 and 19,754 for the eight comparisons. Model building was automated through an iterative process of adding terms to achieve the best balance between the groups [27].

After estimating the PS, we performed PS matching (PSM) and inverse probability of treatment weighting (IPTW). For PSM, a 0.2 standard deviation of the logit of the PS was used as a caliper. The IPTW weight for the average treatment effect for the treated (ATT) was used in this study. Although IPTW can estimate both the average treatment effect (ATE) and ATT, ATT was more appropriate for this study, as some patients receiving warfarin did not meet the standard or reduced dose criteria for patients receiving NOACs [17]. After matching or weighting, balance was evaluated using absolute standardized differences for all covariates [28] and side-by-side boxplots for continuous variables. An absolute standardized difference of <0.1 was considered an acceptable balance. Additionally, a statistical significance test (*p*-value) was applied. If variables did not reach an acceptable level of balance (i.e., standardized difference < 0.1 or *p*-value < 0.05), they were included in the outcome model to be adjusted.

### 2.5. Statistical Analyses—E-Value and Negative Control Outcome

E-values and negative control outcomes were used to detect residual unmeasured confounding [1]. E-values for the hazard ratio (HR) scale for a rare outcome (where outcome < 15%) were calculated for each PS method. In a rare outcome setting, the risk ratio approximates HR [29]. E-values and confidence intervals (CIs) were calculated using a web-based tool [30,31,32]. 

To compare the E-values of the conventional and machine learning methods (hereinafter referred to as E-values from the main analysis), we assessed whether the difference between E-values from the main analysis was larger than the “*meaningful difference Δ*” for each comparison. *Meaningful difference ∆* was defined as the difference between the E-value from the main analysis and the E-value defined as an anchor (reference point). E-values as anchors were derived from the analyses by excluding age and sex from the PS model. The exclusion of age and sex was needed to create a scenario in which a strong unmeasured confounder existed. Age and sex are classical confounders and factors used to predict stroke risk in patients with AF [14,33]. Age- or sex-related variables in the CHA_2_DS_2_-VASc and HAS-BLED scores were also excluded from the PS model. To define one anchor for each comparison, a method that maximized the balance of covariates was chosen. The *meaningful difference ∆* was expressed as an absolute value, as this analysis aimed to assess the magnitude of the impact of a scenario wherein an unmeasured confounder is as powerful as a known measured confounder, such as age or sex. 

If the difference in E-values from the main analysis was larger than the *meaningful difference ∆*, it was considered meaningfully different. If the E-values from the main analysis were not meaningfully different, the percentage of maximum possible coverage (i.e., maximum possible difference in E-values from the main analysis divided by the *meaningful difference ∆*) was calculated to explore the extent to which the method with the highest E-value for each comparison covered *a meaningful difference ∆*. The summarized process of this approach is presented in Table 1.

In this study, the negative control outcomes were pneumonia and urinary tract infections (the definitions are provided in Supplementary Table S3). These outcomes were previously considered in a comparative effectiveness study of NOACs in patients with AF [34]. HRs with 95% CIs were calculated using a Cox proportional hazard model. The proportional hazard assumption was assessed using log–log plots. To ensure comparability between different methods, if the proportional hazard assumption was not met, HRs were not reported.

Statistical significance was set at *p* < 0.05. PS estimation using GBM was performed using R Studio version 1.1.463 (R Foundation for Statistical Computing, Vienna, Austria). To perform GBM, we used the twang (twang: Toolkit for Weighting and Analysis of Nonequivalent Groups) package in R [26]. The R codes are listed in Supplementary Table S4. All other analyses were performed using the SAS Enterprise Guide 6.1 M1 (SAS Institute Inc., Cary, NC, USA).

## 3. Results

### 3.1. Baseline Characteristics and Balance

**Figure 2:**
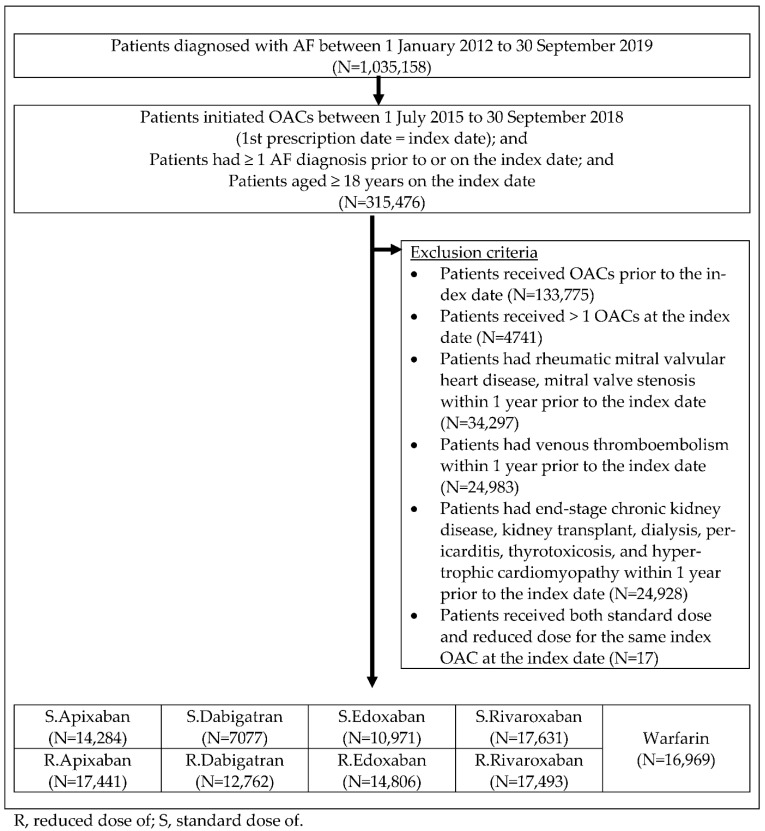
Patient-selection flow.

In total, 129,434 patients with AF who were initiated on OACs were identified (Figure 2). The mean and median follow-up periods for each cohort are presented in Supplementary Table S5. Among all treatment groups, the follow-up period was the longest for the standard and reduced doses of dabigatran.

The baseline characteristics of each index treatment group before and after the application of the PS method are summarized in Supplementary Tables S6–S13. After applying PSM and IPTW, all the baseline characteristics were well balanced. However, although PSM and IPTW were applied, some variables were statistically significant (*p* < 0.05; Supplementary Tables S14–S21). Among these methods, IPTW (GBM) was the only method without statistically significant variables. As all PS methods achieved acceptable balance levels (standardized differences < 0.10), figures with their x-axis (i.e., standardized difference) from 0 to 0.1, are also presented for a closer comparison (Supplementary Figures S1–S8). The conventional approach, especially IPTW (logistic), showed larger standardized differences more often than the other methods. IPTW (GBM) usually showed the smallest standardized difference among all the comparisons. Among the continuous variables used for PS, only age and CHA_2_DS_2_-VASc score are displayed in side-by-side boxplots, as they were the most relevant for effectiveness outcomes (Supplementary Figures S9–S16). PSM (GBM) and IPTW (GBM) produced a more similar distribution between the treatment groups than the conventional approaches.

### 3.2. E-Value 

The E-values were calculated as a sensitivity analysis to assess the results’ robustness to potential residual unmeasured confounding [30]. The E-values for the HRs and for one limit of the CI closest to null were estimated in this study and are presented in Table 2. Additionally, the E-values as an anchor and *meaningful difference ∆* are presented in Table 2. Because the IPTW (GBM) showed a maximized balance among all PS methods, the E-value as an anchor was derived from it. 

Conditional on the measured confounders considered in this study, the range of E-values for adjusted HRs was 1.75–2.70; this indicates that unmeasured confounders associated with exposure and outcome with HRs of at least 1.75–2.70 can nullify the observed HRs; however, this was not the case for weaker unmeasured confounders. The E-values were generally higher in the IPTW (GBM) method than in the other methods (Table 2).

E-values as anchors were generally lower than those from the main analysis, except for Comparison 3 (i.e., the standard dose of dabigatran vs. warfarin). This seems to be related to the fact that Comparison 3 was the only one wherein the NOAC group had a lower age and a lower proportion of women than the warfarin group. Comparisons 1, 3, and 5 showed that the differences in the E-values from the main analysis were larger than the *meaningful difference ∆*. This indicates that the E-values from the main analysis are meaningfully different in Comparisons 1, 3, and 5. The percentages of maximum possible coverage of GBM were 29%, 64%, 30%, and 16% of the *meaningful difference ∆* in Comparisons 2, 4, 7, and 8, respectively (Table 2).

### 3.3. Negative Control Outcome

**Figure 3:**
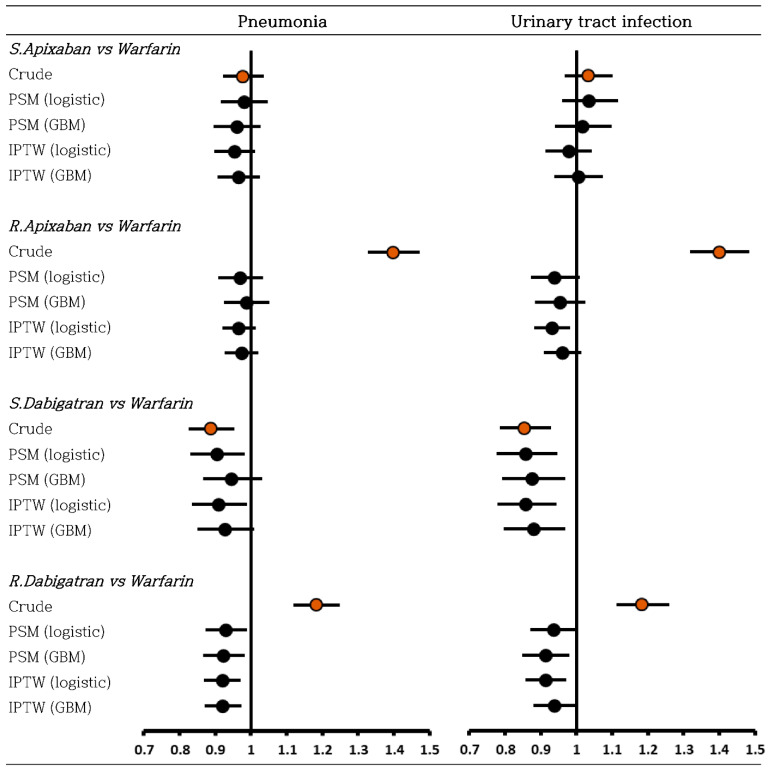

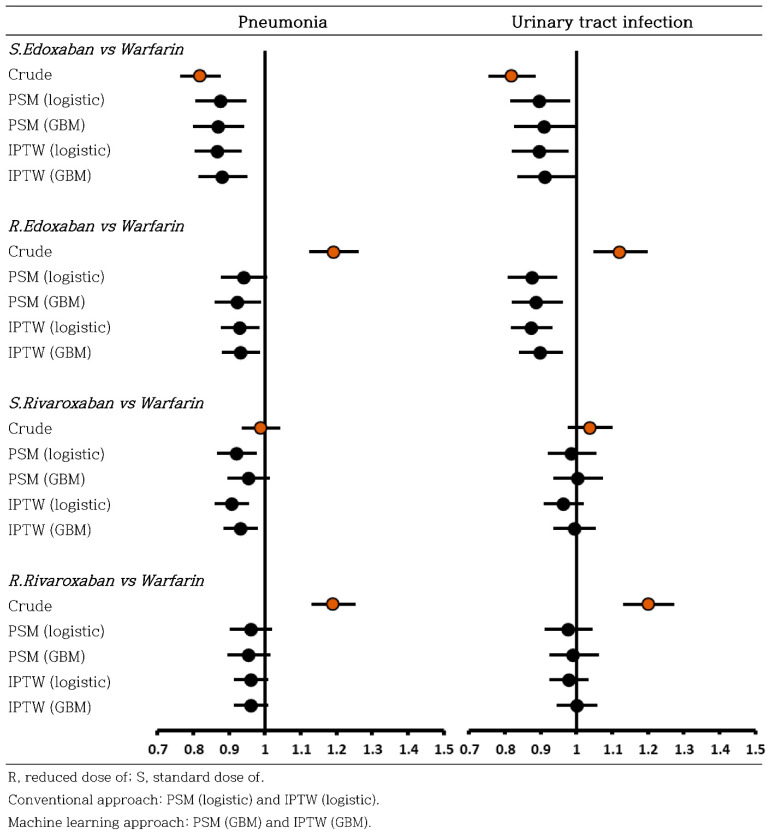
Hazard ratios for negative control outcomes.

For negative control outcome analysis, effect estimates were comparable, and the a priori null hypothesis of neutral associations was generally not rejected in most comparisons (Figure 3). For all comparisons, non-significant HRs were observed slightly more frequently in GBM than in the conventional approach.

## 4. Discussion

Several studies on the comparative effectiveness analysis of OACs in AF patients, including recent studies using the GBM approach, were published worldwide. However, little is known about the advantages or strengths of the GBM approach over simple logistic regression, which is more widely used in this research area. To the best of our knowledge, this study is the first to show that GBM, which is machine learning approach, demonstrated a better ability to balance covariates and had a lower impact of residual confounding than the conventional approach, in the empirical example of comparative effectiveness analysis in patients with AF.

We compared the GBM (machine-learning-based PS) to logistic regression (conventional PS) for balance and residual confounding. Compared with other methods, the IPTW (GBM) showed better balance achievement in terms of standardized difference, statistical significance, and E-value. In the negative control outcome analysis, the smallest number of statistically significant associations was observed in the PSM (GBM). Considering the results on balance ability and residual confounding in this study, GBM would be useful in comparative effectiveness studies in patients with AF. In this study, we attempted to find the GBM-based PS that achieved the best balance through an iterative process. The number of unbalanced covariates was meaningfully reduced in the GBM approaches than in the conventional approaches, even in comparisons of reduced dose NOACs versus warfarin in which it was more difficult to achieve balance.

Our results may not be generalizable to all diseases or exposure–outcome scenarios, as other issues may be found, such as those related to achieving comparability between groups or research questions [35,36]. However, in many settings, the machine learning approach is expected to perform well, especially in balance, owing to its automated computational system [3,18,27]. Nevertheless, findings regarding the balance ability of the conventional versus machine learning approach were inconsistent [35,37,38], which may be due to the different study settings and tuning parameters for machine learning [35]. Alam et al. found that machine-learning-based PS was comparable or inferior to logistic regression in terms of balance, bias, and mean squared error; moreover, considering computational cost, machine learning has no clear advantage [35]. Harder et al. examined some combinations of PS estimation and application methods and found that the performance of machine-learning-based PS could be better when combined with the weighting method [37]. Lee et al. calculated PS weights using logistic regression and machine learning, and found that logistic regression tended to provide a higher number of extreme weights in all scenarios with various degrees of additivity and linearity [38]. It is noteworthy that both conventional and machine learning approaches for deriving PS have advantages and disadvantages. The conventional approach is simple to implement but has key challenges related to selecting an appropriate set of interaction and polynomial terms, as recommended by Rosenbaum et al. and Dehejia et al. [3,18,39,40,41]. Thus, the conventional approach sometimes requires a very time-consuming process to achieve a better balance, which is performed by humans [3]. In contrast, the machine learning approach is known to have the advantage of being able to identify an appropriate set of interaction and polynomial terms using an automated iterative process to achieve the best balance [3]. However, the machine learning approach also has limitations, such as requiring computational storage and time for the iterative process [42]. 

The concept of E-value methodology was introduced and used since 2016 [30,43]. A systematic review reported that a substantial overlap was found in E-values between studies concluding that residual confounding is likely to affect the results and those concluding that residual confounding is unlikely to affect the results [30]. In this review, the median E-values for the point estimate were 1.88, 1.82, and 2.02 for 43 studies concluding that confounding was likely to affect, 348 studies concluding that confounding was unlikely to affect, and 125 studies that provided no comment, respectively [30]. As there are no cut-offs that can be used to interpret the magnitude of residual unmeasured confounding, the result of this new E-value approach should be interpreted cautiously [44]. 

VanderWeele et al. suggested the following considerations when researchers use and interpret E-values: (1) reporting an E-value for estimate and CI, (2) providing discussion regarding specific known unmeasured confounders that could not be accounted for in the analysis, and (3), where possible, comparing E-values to previous studies including confounders that were considered as known unmeasured confounders in the researcher’s study and/or comparing E-values with previous studies that adjusted different combinations of measured confounders from the researcher’s study [44]. We thought that the known unmeasured confounders in this study may be cognitive impairment or frailty; however, it was reported that patients with AF who are comorbid with or without cognitive impairment or frailty experience similar effectiveness (i.e., stroke/transient ischemic attack/non-central nervous system embolism) when using OACs [45]. Thus, although the possibility of some residual unmeasured confounding cannot be completely excluded, the E-values, as a sensitivity analysis in this study, indicate that the study results are likely to be robust to potential residual unmeasured confounding. 

This study has several limitations. First, HIRA claims data were collected for reimbursement and not for research purposes. Thus, coding errors may be found in some claims related to defining covariates or outcomes. To minimize this limitation, codes based on previous studies or clinical expert opinions were used [11,13]. This limitation is expected to have a similar impact on the two groups. 

Second, only logistic regression and GBM were used for conventional and machine-learning-based PS, respectively. Because these two models are increasingly used to derive PS in the field of AF research [6,7,8,17], it can provide insight into a practical and user-friendly comparison based on real experiences. Further research is needed to provide suggestions for which analytical methods are better in each study context. In addition, research comparing machine learning methods with econometric methods is also needed (e.g., the Heckman selection model or instrumental variable method), as these econometric methods were suggested to account for unmeasured confounding [1,46,47]. Given that machine learning/artificial intelligence and big data analysis proved useful for limiting confounding, the concept of this study could be further extended to other research areas, for example, more efficient targeting of candidate genes by minimizing and evaluating confounding in genetic research on cardiovascular diseases [48,49,50].

Third, other PS methods (e.g., stratification or covariate adjustment using PS) were not considered in the comparison. This is because PSM and IPTW were reported to be able to remove more systematic differences in covariates between groups compared with other PS methods (i.e., covariate adjustment using PS or stratification) [4]. Moreover, both PSM and IPTW were recommended for use by researchers for estimating survival outcomes [51]. However, considering the characteristics of PSM and IPTW, the exclusion of unmatched subjects in PSM results can cause loss of generalizability and precision [36]. In addition, PSM or IPTW may not be appropriate in other studies [36]. Thus, researchers should choose an appropriate PS method based on the characteristics of their study [36]. 

Fourth, to compare E-values between methods, *meaningful difference ∆* was defined by the researchers of this study. Only age- and sex-related variables were considered to derive the E-value as an anchor in IPTW (GBM). Therefore, our results should be interpreted based on the definitions used in this study. Despite these limitations, this analysis is meaningful as it provides insights into the possibility of another interpretation approach for E-values.

Fifth, we could not perform a runtime complexity analysis for our GBM, O(k*m), where k is the depth of the tree and m is the number of decision trees in the tree [52,53]. This could provide useful information for evaluating the efficiency of algorithms and the trade-off between performance and runtime [54]. Although we did not perform this analysis, our runtime to estimate PS using GBM was approximately 45 min on the system with an Intel Xeon 4-core 3.00 GHz CPU and 8 GB RAM, whereas it was 8–10 min when using conventional logistic regression. Future machine learning studies should consider complexity analysis to help readers find a good balance between performance and runtime. Although a longer running time was needed to perform PS using GBM, ML has a useful feature in that it can identify the PS model that provides the best balance [3]; this benefit may outweigh the longer running time needed for ML because of advancements in computer processing power.

## 5. Conclusions

This study compared the ability to balance baseline covariates and explored the impact of residual confounding between conventional and machine-learning-based PS in the context of a comparative effectiveness study into patients with AF. We showed that GBM provided a better ability to balance covariates and had a lower impact on residual confounding, compared with the conventional approach, in the empirical example of comparative effectiveness analysis in patients with AF. Considering that the PS method is widely used, this study highlights the need for a more suitable and efficient adjustment method. This study serves as a basis for developing more advanced practical applications of machine learning and artificial intelligence in future observational studies.

## Figures and Tables

**Table 1:** Summarized process for comparing E-values in this study.

(1) Calculate E-values from main analysis (E-values from conventional and machine learning approaches). (2) Calculate E-value as an anchor (E-value derived from age/sex excluded PS model) for each comparison. (3) Calculate *meaningful difference Δ*: E-value from main analysis (1) − E-value as an anchor (2) = (1) − (2). (4) Calculate difference in E-values from main analysis (e.g., E-value from IPTW (GBM) − E-value from IPTW (logistic)). (5) If difference in E-values from main analysis (4) ≥ *meaningful difference Δ* (3), E-values from main analysis are meaningfully different. (6) If difference in E-values from main analysis (4) < *meaningful difference Δ* (3), E-values from main analysis are not meaningfully different. (7) Calculate maximum possible coverage for the comparisons for (6). $maximum\;possible\;coverage=\frac{\left(maximum\;E-value\;from\;main\;analysis\right)-\left(minimum\;E-value\;from\;main\;analysis\right)}{meaningful\;difference\;\Delta }$

**Table 2:** E-values of the estimated hazard ratios for stroke or systemic embolism.

Comparisons	E-value for the Hazard Ratios ^(1–3)^ (E-Value for the Limit of Confidence Interval Closest to the Null)				E-Value as an Anchor ^(4)^	Meaningful Difference *Δ* ^(5)^	Maximum Possible Coverage (%) ^(6)^
	PSM (Logistic)	PSM (GBM)	IPTW (Logistic)	IPTW (GBM)	IPTW (GBM)		
Comparison 1	2.24 (1.92)	2.23 (1.90)	2.32 (2.04)	2.35 (2.07)	2.32	0.03	-
Comparison 2	1.96 (1.66)	2.01 (1.71)	2.00 (1.78)	2.08 (1.85)	1.67	0.41	29
Comparison 3	2.13 (1.76)	2.19 (1.81)	2.19 (1.82)	2.30 (1.93)	2.36	0.06	-
Comparison 4	1.75 (1.47)	1.89 (1.60)	1.89 (1.64)	1.93 (1.68)	1.65	0.28	64
Comparison 5	2.45 (2.05)	2.27 (1.88)	2.52 (2.13)	2.43 (2.05)	2.42	0.01	-
Comparison 6	2.70 (2.32)	2.67 (2.28)	2.53 (2.21)	2.56 (2.24)	2.10	0.46	37
Comparison 7	2.01 (1.73)	2.04 (1.75)	2.02 (1.78)	2.04 (1.80)	1.94	0.10	30
Comparison 8	2.05 (1.76)	2.08 (1.78)	2.04 (1.80)	2.10 (1.85)	1.72	0.38	16

Comparison 1, standard dose of apixaban vs warfarin; Comparison 2, reduced dose of apixaban vs warfarin; Comparison 3, standard dose of dabigatran vs warfarin; Comparison 4, reduced dose of dabigatran vs warfarin; Comparison 5, standard dose of edoxaban vs warfarin; Comparison 6, reduced dose of edoxaban vs warfarin; Comparison 7, standard dose of rivaroxaban vs warfarin; Comparison 8, reduced dose of rivaroxaban vs warfarin. ^(1)^ Conventional approach: PSM (logistic) and IPTW (logistic). ^(2)^ Machine learning approach: PSM (GBM) and IPTW (GBM). ^(3)^ Larger E-value indicates that the stronger unmeasured confounder associations can nullify the observed hazard ratios. ^(4)^ E-values derived from analysis that excludes age and sex variables from the PS model. ^(5)^ *Meaningful difference Δ* was defined by the researchers of this study as the difference between E-value from main analysis and E-value defined as an anchor. Interpretation under the definition used in this study is needed. ^(6)^ Maximum possible coverage (%) = [(maximum E-value from main analysis) − (minimum E-value from main analysis)]/(*meaningful difference Δ*) × 100. It was calculated for the comparisons that E-values from main analysis were not meaningfully different.

## Data Availability

The data used in this study are available at https://opendata.hira.or.kr/ (accessed on 27 May 2020) with permission from the Health Insurance Review and Assessment Service. Data were provided by the Health Insurance Review and Assessment Service after reviewing the researcher’s request for academic purposes.

## Supplementary Materials

The following supporting information can be downloaded at: https://www.mdpi.com/article/10.3390/ijerph191912916/s1. Figure S1: Absolute standardized differences in comparison 1; Figure S2: Absolute standardized differences in comparison 2; Figure S3: Absolute standardized differences in comparison 3; Figure S4: Absolute standardized differences in comparison 4; Figure S5: Absolute standardized differences in comparison 5; Figure S6: Absolute standardized differences in comparison 6; Figure S7: Absolute standardized differences in comparison 7; Figure S8: Absolute standardized differences in comparison 8; Figure S9: Side-by-side boxplot in comparison 1; Figure S10: Side-by-side boxplot in comparison 2; Figure S11: Side-by-side boxplot in comparison 3; Figure S12: Side-by-side boxplot in comparison 4; Figure S13: Side-by-side boxplot in comparison 5; Figure S14: Side-by-side boxplot in comparison 6; Figure S15: Side-by-side boxplot in comparison 7; Figure S16: Side-by-side boxplot in comparison 8; Table S1: Definitions for stroke or systemic embolism; Table S2: Propensity score variables during pre-index period; Table S3: Definitions for negative control outcomes; Table S4: R codes for generalized boosted model to estimate propensity score; Table S5: Length of follow-up period in each comparison; Table S6: Baseline characteristics for comparison 1 (standard dose of apixaban vs warfarin); Table S7: Baseline characteristics for comparison 2 (reduced dose of apixaban vs warfarin); Table S8: Baseline characteristics for comparison 3 (standard dose of dabigatran vs warfarin); Table S9: Baseline characteristics for comparison 4 (reduced dose of dabigatran vs warfarin); Table S10: Baseline characteristics for comparison 5 (standard dose of edoxaban vs warfarin); Table S11: Baseline characteristics for comparison 6 (reduced dose of edoxaban vs warfarin); Table S12: Baseline characteristics for comparison 7 (standard dose of rivaroxaban vs warfarin); Table S13: Baseline characteristics for comparison 8 (reduced dose of rivaroxaban vs warfarin); Table S14: Absolute standardized difference of baseline characteristics in comparison 1; Table S15: Absolute standardized difference of baseline characteristics in comparison 2; Table S16: Absolute standardized difference of baseline characteristics in comparison 3; Table S17: Absolute standardized difference of baseline characteristics in comparison 4; Table S18: Absolute standardized difference of baseline characteristics in comparison 5; Table S19: Absolute standardized difference of baseline characteristics in comparison 6; Table S20: Absolute standardized difference of baseline characteristics in comparison 7; Table S21: Absolute standardized difference of baseline characteristics in comparison 8.
