# Supplementary material for: Impact of Integrating Machine Learning in Comparative Effectiveness Research of Oral Anticoagulants in Patients with Atrial Fibrillation
Source: Int J Environ Res Public Health. 2022 Oct 9;19(19):12916. doi: 10.3390/ijerph191912916 (PMC9566283; doi:10.3390/ijerph191912916)
Supplement: Supplementary file 1 [file ijerph-19-12916-s001.zip › ijerph-1918711-supplementary.pdf]

Supplementary Table S1. Definitions for stroke or systemic embolism

Outcome	Definition
Stroke	
Ischemic stroke	Hospitalization with diagnosis codes of I63 and I64 with brain CT/MRI record
Hemorrhagic stroke	Hospitalization with diagnosis codes of I60, I61, and I62 with brain CT/MRI record
Systemic embolism	
Systemic embolism	Hospitalization with diagnosis codes of I74 with any CT/MRI record

Supplementary Table S2. Propensity score variables during pre-index period

Variables	Definition
Age	Age measured in years at the index date
Sex	Sex as reported in data (male/female)
Insurance	Insurance type at the index date (National Health Insurance/Medical aid)
CHA_2DS_2-VASc	CHA_2DS_2-VASc score with characteristics during the pre-index period
HAS-BLED (modified)*	HAS-BLED score with characteristics during the pre-index period
Charlson Comorbidity Index	Charlson Comorbidity Index with characteristics during the pre-index period
Chronic pulmonary disease	≥ 1 claim with diagnosis codes of chronic pulmonary disease during the pre-index period (yes/no – diagnosis codes: I27.8, I27.9, J40–J47, J60–J67, J68.4, J70.1, J70.3)
Myocardial infarction	≥ 1 claim with KCD codes of myocardial infarction during the pre-index period (yes/no – diagnosis codes: I21, I22, I25.2)
Peripheral vascular disease	≥ 1 claim with diagnosis codes of peripheral vascular disease during the pre-index period (yes/no – diagnosis codes: I70, I71, I73.1, I73.8, I73.9, I77.1, I79.0, I79.2, K55.1, K55.8, K55.9, Z95.8, Z95.9)
Renal disease	≥ 1 claim with diagnosis codes of renal disease during

Variables	Definition
	the pre-index period (yes/no – diagnosis codes: I12.0, I13.1, N03.2-N03.7, N05.2-N05.7, N18, N19, N25.0, Z49.0-Z49.2, Z94.0, Z99.2)
NSAIDs use	≥ 1 claim with NSAIDs medication codes during the pre-index period (yes/no)
Antiplatelets use	≥ 1 claim with antiplatelets medication codes during the pre-index period (yes/no)
Statins use	≥ 1 claim with statins medication codes during the pre-index period (yes/no)
Antiarrythmics use	≥ 1 claim with antiarrythmics medication codes during the pre-index period (yes/no)
Proton pump inhibitors use	≥ 1 claim with proton pump inhibitors medication codes during the pre-index period (yes/no)
H_2-receptor antagonists use	≥ 1 claim with H_2-receptor antagonists medication codes during the pre-index period (yes/no)
Digoxin use	≥ 1 claim with digoxin medication codes during the pre-index period (yes/no)

* In Health Insurance Review & Assessment Service (HIRA) database, labile international normalized ratio in HAS-BLED score is not available and it is not applicable to apixaban/dabigatran/edoxaban/rivaroxaban groups. Thus, labile international normalized ratio variable was excluded and modified HAS-BLED score that has a maximum value of 8 was used.

Supplementary Table S3. Definitions for negative control outcomes

Variables	Definition
Pneumonia	≥ 1 claim with diagnosis codes of pneumonia during the follow-up period (yes/no – diagnosis codes: J12, J13, J14, J15, J16, J17, J18)
Urinary tract infection	≥ 1 claim with diagnosis codes of urinary tract infection during the follow-up period (yes/no – diagnosis codes: N390, N9989)

Supplementary Table S4. R codes for generalized boosted model to estimate propensity score

```
install.packages("twang")
library(twang)

#sub1 - standard apixaban versus warfarin
ps.sub1 <- ps(trt ~ age + age2 + sex + insg + cha + has + cci +
                 ch1 + ch2 + ch3 + ch4 + ch5 + ch6 + ch7 + ch8 +
                 hs1 + hs2 + hs3 + hs4 + hs5 + hs6 + hs7 + hs8 +
                 cpd + mi + pvd + rd + nsaid + antip + statin + antiarr +
                 ppi + h2b + digox,
               data=sub1,
               n.trees=20000, interaction.depth=2,
               shrinkage=0.01, perm.test.iters=0,
               stop.method=c("es.max"),
               estimand="ATT", verbose=FALSE)

summary(ps.sub1)

#sub2 - reduced apixaban versus warfarin
ps.sub2 <- ps(trt ~ age + age2 + sex + insg + cha + has + cci +
                 ch1 + ch2 + ch3 + ch4 + ch5 + ch6 + ch7 + ch8 +
                 hs1 + hs2 + hs3 + hs4 + hs5 + hs6 + hs7 + hs8 +
                 cpd + mi + pvd + rd + nsaid + antip + statin + antiarr +
                 ppi + h2b + digox,
               data=sub2,
               n.trees=20000, interaction.depth=2,
               shrinkage=0.01, perm.test.iters=0,
               stop.method=c("es.max"),
               estimand="ATT", verbose=FALSE)

summary(ps.sub2)

#sub3 - standard dabigatran versus warfarin
ps.sub3 <- ps(trt ~ age + age2 + sex + insg + cha + has + cci +
                 ch1 + ch2 + ch3 + ch4 + ch5 + ch6 + ch7 + ch8 +
                 hs1 + hs2 + hs3 + hs4 + hs5 + hs6 + hs7 + hs8 +
                 cpd + mi + pvd + rd + nsaid + antip + statin + antiarr +
                 ppi + h2b + digox,
```

```
                     data=sub3,
                     n.trees=20000, interaction.depth=2,
                     shrinkage=0.01, perm.test.iters=0,
                     stop.method=c("es.max"),
                     estimand="ATT", verbose=FALSE)

summary(ps.sub3)

#sub4 - reduced dabigatran versus warfarin
ps.sub4 <- ps(trt ~ age + age2 + sex + insg + cha + has + cci +
                       ch1 + ch2 + ch3 + ch4 + ch5 + ch6 + ch7 + ch8 +
                       hs1 + hs2 + hs3 + hs4 + hs5 + hs6 + hs7 + hs8 +
                       cpd + mi + pvd + rd + nsaid + antip + statin + antiarr +
                       ppi + h2b + digox,
                     data=sub4,
                     n.trees=20000, interaction.depth=2,
                     shrinkage=0.01, perm.test.iters=0,
                     stop.method=c("es.max"),
                     estimand="ATT", verbose=FALSE)

summary(ps.sub4)

#sub5 - standard edoxaban versus warfarin
ps.sub5 <- ps(trt ~ age + age2 + sex + insg + cha + has + cci +
                       ch1 + ch2 + ch3 + ch4 + ch5 + ch6 + ch7 + ch8 +
                       hs1 + hs2 + hs3 + hs4 + hs5 + hs6 + hs7 + hs8 +
                       cpd + mi + pvd + rd + nsaid + antip + statin + antiarr +
                       ppi + h2b + digox,
                     data=sub5,
                     n.trees=20000, interaction.depth=2,
                     shrinkage=0.01, perm.test.iters=0,
                     stop.method=c("es.max"),
                     estimand="ATT", verbose=FALSE)

summary(ps.sub5)

#sub6 - reduced edoxaban versus warfarin
ps.sub6 <- ps(trt ~ age + age2 + sex + insg + cha + has + cci +
                       ch1 + ch2 + ch3 + ch4 + ch5 + ch6 + ch7 + ch8 +
```

```
                hs1 + hs2 + hs3 + hs4 + hs5 + hs6 + hs7 + hs8 +
                cpd + mi + pvd + rd + nsaid + antip + statin + antiarr +
                ppi + h2b + digox,
              data=sub6,
              n.trees=20000, interaction.depth=2,
              shrinkage=0.01, perm.test.iters=0,
              stop.method=c("es.max"),
              estimand="ATT", verbose=FALSE)

summary(ps.sub6)

#sub7 - standard rivaroxaban versus warfarin
ps.sub7 <- ps(trt ~ age + age2 + sex + insg + cha + has + cci +
                ch1 + ch2 + ch3 + ch4 + ch5 + ch6 + ch7 + ch8 +
                hs1 + hs2 + hs3 + hs4 + hs5 + hs6 + hs7 + hs8 +
                cpd + mi + pvd + rd + nsaid + antip + statin + antiarr +
                ppi + h2b + digox,
              data=sub7,
              n.trees=20000, interaction.depth=2,
              shrinkage=0.01, perm.test.iters=0,
              stop.method=c("es.max"),
              estimand="ATT", verbose=FALSE)

summary(ps.sub7)

#sub8 - reduced rivaroxaban versus warfarin
ps.sub8 <- ps(trt ~ age + age2 + sex + insg + cha + has + cci +
                ch1 + ch2 + ch3 + ch4 + ch5 + ch6 + ch7 + ch8 +
                hs1 + hs2 + hs3 + hs4 + hs5 + hs6 + hs7 + hs8 +
                cpd + mi + pvd + rd + nsaid + antip + statin + antiarr +
                ppi + h2b + digox,
              data=sub8,
              n.trees=20000, interaction.depth=2,
              shrinkage=0.01, perm.test.iters=0,
              stop.method=c("es.max"),
              estimand="ATT", verbose=FALSE)

summary(ps.sub8)
```

```
summary(ps.sub1$gbm.obj,
        n.trees=ps.sub1$desc$es.max.ATT$n.trees,
        plot=TRUE)
plot(ps.sub1, plots=1)
plot(ps.sub1, plots=3)
plot(ps.sub1, plots=4)
plot(ps.sub1, plots=5)

summary(ps.sub2$gbm.obj,
        n.trees=ps.sub2$desc$es.max.ATT$n.trees,
        plot=TRUE)
plot(ps.sub2, plots=1)
plot(ps.sub2, plots=3)
plot(ps.sub2, plots=4)
plot(ps.sub2, plots=5)

summary(ps.sub3$gbm.obj,
        n.trees=ps.sub3$desc$es.max.ATT$n.trees,
        plot=TRUE)
plot(ps.sub3, plots=1)
plot(ps.sub3, plots=3)
plot(ps.sub3, plots=4)
plot(ps.sub3, plots=5)

summary(ps.sub4$gbm.obj,
        n.trees=ps.sub4$desc$es.max.ATT$n.trees,
        plot=TRUE)
plot(ps.sub4, plots=1)
plot(ps.sub4, plots=3)
plot(ps.sub4, plots=4)
plot(ps.sub4, plots=5)

summary(ps.sub5$gbm.obj,
        n.trees=ps.sub5$desc$es.max.ATT$n.trees,
        plot=TRUE)
plot(ps.sub5, plots=1)
plot(ps.sub5, plots=3)
plot(ps.sub5, plots=4)
plot(ps.sub5, plots=5)
```

```
summary(ps.sub6$gbm.obj,
        n.trees=ps.sub6$desc$es.max.ATT$n.trees,
        plot=TRUE)
plot(ps.sub6, plots=1)
plot(ps.sub6, plots=3)
plot(ps.sub6, plots=4)
plot(ps.sub6, plots=5)

summary(ps.sub7$gbm.obj,
        n.trees=ps.sub7$desc$es.max.ATT$n.trees,
        plot=TRUE)
plot(ps.sub7, plots=1)
plot(ps.sub7, plots=3)
plot(ps.sub7, plots=4)
plot(ps.sub7, plots=5)

summary(ps.sub8$gbm.obj,
        n.trees=ps.sub8$desc$es.max.ATT$n.trees,
        plot=TRUE)
plot(ps.sub8, plots=1)
plot(ps.sub8, plots=3)
plot(ps.sub8, plots=4)
plot(ps.sub8, plots=5)

bal.table(ps.sub1, digits=3)
bal.table(ps.sub2, digits=3)
bal.table(ps.sub3, digits=3)
bal.table(ps.sub4, digits=3)
bal.table(ps.sub5, digits=3)
bal.table(ps.sub6, digits=3)
bal.table(ps.sub7, digits=3)
bal.table(ps.sub8, digits=3)
```

Supplementary Table S5. Length of follow-up period in each comparison

Cohorts	Follow-up periods (days)	
	Mean (SD)	Median (IQR)
Standard dose of apixaban	851 (370)	814 (618)
Reduced dose of apixaban	794 (388)	768 (592)
Standard dose of dabigatran	1037 (364)	1085 (595)
Reduced dose of dabigatran	1013 (391)	1070 (622)
Standard dose of edoxaban	712 (252)	685 (399)
Reduced dose of edoxaban	691 (271)	670 (412)
Standard dose of rivaroxaban	951 (395)	971 (682)
Reduced dose of rivaroxaban	899 (391)	902 (669)
Warfarin	975 (406)	1033 (647)

Supplementary Table S6. Baseline characteristics for comparison 1 (standard dose of apixaban vs warfarin)

Variables	Crude		PSM (logistic)		PSM (GBM)		IPTW (logistic)		IPTW (GBM)	
	S.Api (N=14,284)	Warfarin (N=16,969)	S.Api (N=11,504)	Warfarin (N=11,504)	S.Api (N=10,648)	Warfarin (N=10,648)	S.Api (N=14,284)	Warfarin (N=14,318)	S.Api (N=14,284)	Warfarin (N=14,010)
	Mean/%	Mean/%	Mean/%	Mean/%	Mean/%	Mean/%	Mean/%	Mean/%	Mean/%	Mean/%
Age	68.50	67.16	68.12	68.34	68.41	68.45	68.50	68.59	68.50	68.52
CCI	3.55	3.49	3.54	3.54	3.64	3.64	3.55	3.63	3.55	3.55
CHA_2DS_2-VASc	4.24	3.88	4.12	4.13	4.22	4.23	4.24	4.31	4.24	4.25
HAS-BLED	3.45	3.28	3.40	3.40	3.46	3.46	3.45	3.49	3.45	3.46
Female	37.49	35.39	36.48	36.79	36.85	36.40	37.49	37.43	37.49	37.19
NHI	94.64	93.30	94.17	94.13	94.00	93.93	94.64	94.44	94.64	94.60
Medical aid	5.36	6.70	5.83	5.87	6.00	6.07	5.36	5.56	5.36	5.40
CHF	36.85	37.40	37.00	37.24	38.61	38.35	36.85	37.11	36.85	36.34
HTN	85.71	78.89	83.71	84.09	85.34	85.59	85.71	86.32	85.71	85.86
DM	47.01	42.66	45.79	46.08	47.72	48.21	47.01	48.38	47.01	47.44
IS	31.98	24.06	29.19	28.52	28.91	29.67	31.98	33.55	31.98	32.26
Bleeding	19.94	26.12	22.29	22.34	23.52	22.94	19.94	20.29	19.94	19.82
CPD	42.31	44.34	43.18	43.79	44.84	44.76	42.31	42.99	42.31	42.27
MI	4.71	6.82	5.48	5.29	5.83	5.74	4.71	4.80	4.71	4.76
PVD	23.52	24.05	24.06	23.88	24.79	24.66	23.52	23.75	23.52	23.50
RD	3.12	7.67	3.85	3.96	4.12	3.78	3.12	3.16	3.12	3.09
NSAIDs	99.17	98.56	99.03	99.01	99.07	99.00	99.17	99.16	99.17	99.22
Antiplatelets	82.05	82.26	82.62	82.38	83.57	83.23	82.05	82.38	82.05	82.29
Statins	75.11	67.96	72.95	73.28	73.65	73.73	75.11	75.71	75.11	75.45
Antiarrhythmics	76.26	73.99	75.50	75.35	75.04	74.75	76.26	76.12	76.26	76.05
PPI	83.14	81.31	82.31	82.68	82.87	82.64	83.14	83.25	83.14	83.28
H2RA	95.67	95.43	95.61	95.69	95.60	95.79	95.67	95.64	95.67	95.65
Digoxin	27.95	36.43	31.31	31.44	33.71	33.45	27.95	28.48	27.95	28.30

CCI, Charson Comorbidity Index; CHA_2DS_2-VASc, congestive heart failure, hypertension, age ≥75 years, diabetes mellitus, stroke, vascular disease, age 65–74 years, and sex; CHF, congestive heart failure; CPD, chronic pulmonary disease; DM, diabetes mellitus; HAS-BLED, hypertension, abnormal renal and liver function, stroke, bleeding, labile international normalized ratio, elderly, drugs, or alcohol; H2RA, H_2-receptor antagonist; HTN, hypertension; IS, ischemic stroke; IPTW, inverse probability of treatment weighting; MI, myocardial infarction; NHI, National Health Insurance; PPI, proton pump inhibitor; PSM, propensity score matching; PVD, peripheral vascular disease; RD, renal disease; S.Api, standard dose of apixaban.

Supplementary Table S7. Baseline characteristics for comparison 2 (reduced dose of apixaban vs warfarin)

Variables	Crude		PSM (logistic)		PSM (GBM)		IPTW (logistic)		IPTW (GBM)	
	R.Api (N=17,441)	Warfarin (N=16,969)	R.Api (N=10,435)	Warfarin (N=10,435)	R.Api (N=10,211)	Warfarin (N=10,211)	R.Api (N=17,441)	Warfarin (N=17,553)	R.Api (N=17,441)	Warfarin (N=17,034)
	Mean/%	Mean/%	Mean/%	Mean/%	Mean/%	Mean/%	Mean/%	Mean/%	Mean/%	Mean/%
Age	77.70	67.16	74.67	74.51	74.52	74.64	77.70	77.83	77.70	77.62
CCI	4.18	3.49	4.07	4.09	4.20	4.19	4.18	4.32	4.18	4.17
CHA_2DS_2-VASc	5.26	3.88	4.86	4.85	4.95	4.98	5.26	5.36	5.26	5.25
HAS-BLED	3.79	3.28	3.73	3.75	3.79	3.80	3.79	3.83	3.79	3.79
Female	54.42	35.39	47.04	46.26	47.09	47.43	54.42	55.70	54.42	54.50
NHI	91.64	93.30	91.86	91.93	91.69	91.70	91.64	91.27	91.64	91.55
Medical aid	8.36	6.70	8.14	8.07	8.31	8.30	8.36	8.73	8.36	8.45
CHF	44.91	37.40	41.76	41.27	42.35	42.65	44.91	45.84	44.91	44.45
HTN	88.80	78.89	86.52	86.70	88.35	88.63	88.80	89.96	88.80	88.90
DM	49.13	42.66	48.72	48.93	50.97	50.93	49.13	51.13	49.13	49.16
IS	31.83	24.06	30.00	30.17	31.47	32.06	31.83	33.58	31.83	31.98
Bleeding	27.30	26.12	28.22	28.57	29.57	29.18	27.30	28.17	27.30	27.45
CPD	50.71	44.34	50.21	50.48	51.18	51.41	50.71	51.26	50.71	50.65
MI	7.90	6.82	7.89	7.73	8.38	8.25	7.90	8.65	7.90	8.19
PVD	28.42	24.05	28.06	28.51	28.88	28.96	28.42	28.62	28.42	28.46
RD	8.53	7.67	9.73	9.86	10.46	10.40	8.53	9.27	8.53	8.54
NSAIDs	98.86	98.56	98.81	98.66	98.64	98.62	98.86	98.71	98.86	98.87
Antiplatelets	84.90	82.26	84.64	85.02	86.02	86.03	84.90	85.59	84.90	85.08
Statins	71.77	67.96	71.92	72.43	73.70	74.05	71.77	73.22	71.77	72.21
Antiarrhythmics	70.71	73.99	71.21	71.14	71.19	70.99	70.71	70.35	70.71	70.69
PPI	84.04	81.31	83.96	83.82	83.85	84.02	84.04	84.41	84.04	84.33
H2RA	96.57	95.43	96.35	96.58	96.39	96.59	96.57	96.64	96.57	96.58
Digoxin	39.21	36.43	39.89	39.86	41.21	40.51	39.21	39.97	39.21	39.56

CCI, Charson Comorbidity Index; CHA_2DS_2-VASc, congestive heart failure, hypertension, age ≥75 years, diabetes mellitus, stroke, vascular disease, age 65–74 years, and sex; CHF, congestive heart failure; CPD, chronic pulmonary disease; DM, diabetes mellitus; HAS-BLED, hypertension, abnormal renal and liver function, stroke, bleeding, labile international normalized ratio, elderly, drugs, or alcohol; H2RA, H_2-receptor antagonist; HTN, hypertension; IS, ischemic stroke; IPTW, inverse probability of treatment weighting; MI, myocardial infarction; NHI, National Health Insurance; PPI, proton pump inhibitor; PSM, propensity score matching; PVD, peripheral vascular disease; RD, renal disease; R.Api, reduced dose of apixaban.

Supplementary Table S8. Baseline characteristics for comparison 3 (standard dose of dabigatran vs warfarin)

Variables	Crude		PSM (logistic)		PSM (GBM)		IPTW (logistic)		IPTW (GBM)	
	S.Dabi (N=7,077)	Warfarin (N=16,969)	S.Dabi (N=6,934)	Warfarin (N=6,934)	S.Dabi (N=6,586)	Warfarin (N=6,586)	S.Dabi (N=7,077)	Warfarin (N=7,093)	S.Dabi (N=7,077)	Warfarin (N=6,956)
	Mean/%	Mean/%	Mean/%	Mean/%	Mean/%	Mean/%	Mean/%	Mean/%	Mean/%	Mean/%
Age	66.57	67.16	66.56	66.93	66.82	66.75	66.57	66.79	66.57	66.64
CCI	3.42	3.49	3.41	3.47	3.43	3.40	3.42	3.48	3.42	3.42
CHA_2DS_2-VASc	4.12	3.88	4.09	4.16	4.11	4.10	4.12	4.18	4.12	4.12
HAS-BLED	3.38	3.28	3.37	3.41	3.38	3.38	3.38	3.42	3.38	3.38
Female	32.26	35.39	32.30	32.79	33.54	32.66	32.26	32.64	32.26	32.24
NHI	94.38	93.30	94.35	94.33	94.17	94.38	94.38	94.15	94.38	94.31
Medical aid	5.62	6.70	5.65	5.67	5.83	5.62	5.62	5.85	5.62	5.69
CHF	36.46	37.40	36.28	37.16	36.97	36.85	36.46	36.51	36.46	35.92
HTN	84.94	78.89	84.73	85.71	85.42	86.44	84.94	85.56	84.94	85.26
DM	48.41	42.66	48.01	48.82	48.36	48.53	48.41	49.31	48.41	48.63
IS	36.33	24.06	35.30	35.28	33.15	33.68	36.33	37.35	36.33	36.39
Bleeding	17.22	26.12	17.51	17.42	18.36	17.75	17.22	17.39	17.22	17.03
CPD	40.02	44.34	40.29	41.33	41.31	41.00	40.02	40.42	40.02	40.12
MI	3.42	6.82	3.49	3.49	3.66	3.17	3.42	3.46	3.42	3.47
PVD	23.41	24.05	23.44	24.47	24.01	24.11	23.41	23.62	23.41	23.23
RD	2.13	7.67	2.18	2.31	2.29	1.90	2.13	2.18	2.13	2.16
NSAIDs	98.98	98.56	98.98	99.05	98.95	98.92	98.98	98.97	98.98	99.01
Antiplatelets	85.59	82.26	85.39	85.84	85.29	85.82	85.59	85.94	85.59	85.95
Statins	77.58	67.96	77.17	77.88	76.33	77.39	77.58	77.69	77.58	77.45
Antiarrhythmics	72.95	73.99	73.15	72.99	73.72	73.35	72.95	73.10	72.95	72.83
PPI	82.89	81.31	82.85	82.72	82.89	82.69	82.89	83.09	82.89	83.01
H2RA	95.55	95.43	95.50	95.70	95.54	95.37	95.55	95.55	95.55	95.50
Digoxin	29.65	36.43	30.03	30.05	31.28	30.93	29.65	29.94	29.65	29.97

CCI, Charson Comorbidity Index; CHA_2DS_2-VASc, congestive heart failure, hypertension, age ≥75 years, diabetes mellitus, stroke, vascular disease, age 65–74 years, and sex; CHF, congestive heart failure; CPD, chronic pulmonary disease; DM, diabetes mellitus; HAS-BLED, hypertension, abnormal renal and liver function, stroke, bleeding, labile international normalized ratio, elderly, drugs, or alcohol; H2RA, H_2-receptor antagonist; HTN, hypertension; IS, ischemic stroke; IPTW, inverse probability of treatment weighting; MI, myocardial infarction; NHI, National Health Insurance; PPI, proton pump inhibitor; PSM, propensity score matching; PVD, peripheral vascular disease; RD, renal disease; S.Dabi, standard dose of dabigatran.

Supplementary Table S9. Baseline characteristics for comparison 4 (reduced dose of dabigatran vs warfarin)

Variables	Crude		PSM (logistic)		PSM (GBM)		IPTW (logistic)		IPTW (GBM)	
	R.Dabi (N=12,762)	Warfarin (N=16,969)	R.Dabi (N=10,451)	Warfarin (N=10,451)	R.Dabi (N=10,045)	Warfarin (N=10,045)	R.Dabi (N=12,762)	Warfarin (N=12,802)	R.Dabi (N=12,762)	Warfarin (N=12,590)
	Mean/%	Mean/%	Mean/%	Mean/%	Mean/%	Mean/%	Mean/%	Mean/%	Mean/%	Mean/%
Age	74.35	67.16	73.46	73.42	73.27	73.53	74.35	74.41	74.35	74.32
CCI	3.74	3.49	3.76	3.82	3.85	3.88	3.74	3.83	3.74	3.75
CHA_2DS_2-VASc	4.88	3.88	4.71	4.72	4.78	4.82	4.88	4.95	4.88	4.88
HAS-BLED	3.66	3.28	3.64	3.67	3.68	3.71	3.66	3.69	3.66	3.67
Female	47.04	35.39	44.57	44.28	45.50	45.43	47.04	47.93	47.04	47.10
NHI	91.77	93.30	92.08	91.85	91.90	91.99	91.77	91.58	91.77	91.82
Medical aid	8.23	6.70	7.92	8.15	8.10	8.01	8.23	8.42	8.23	8.18
CHF	39.37	37.40	39.18	38.92	39.82	39.67	39.37	39.51	39.37	38.85
HTN	89.15	78.89	87.11	87.51	87.98	88.26	89.15	89.70	89.15	89.14
DM	47.78	42.66	46.84	47.89	49.17	49.75	47.78	49.44	47.78	48.32
IS	30.89	24.06	29.81	30.08	31.19	31.74	30.89	32.65	30.89	31.31
Bleeding	21.47	26.12	24.13	25.38	24.95	25.69	21.47	21.79	21.47	21.44
CPD	46.19	44.34	47.17	48.17	47.83	48.64	46.19	46.49	46.19	46.28
MI	5.32	6.82	6.14	6.28	6.23	6.43	5.32	5.48	5.32	5.43
PVD	27.56	24.05	27.47	27.52	27.87	27.58	27.56	27.84	27.56	27.73
RD	3.77	7.67	4.60	4.88	4.77	4.62	3.77	3.84	3.77	3.80
NSAIDs	98.95	98.56	98.84	98.80	98.76	98.78	98.95	98.86	98.95	98.89
Antiplatelets	86.51	82.26	85.85	85.52	85.92	86.43	86.51	86.63	86.51	86.46
Statins	74.28	67.96	72.75	72.74	74.05	74.42	74.28	75.33	74.28	74.56
Antiarrhythmics	70.54	73.99	72.12	71.09	71.11	71.13	70.54	70.19	70.54	70.28
PPI	84.38	81.31	83.67	83.60	84.02	83.79	84.38	84.67	84.38	84.48
H2RA	96.90	95.43	96.56	96.65	96.72	96.64	96.60	96.95	96.90	96.83
Digoxin	37.06	36.43	38.22	38.92	39.13	39.75	37.06	37.34	37.06	37.21

CCI, Charson Comorbidity Index; CHA_2DS_2-VASc, congestive heart failure, hypertension, age ≥75 years, diabetes mellitus, stroke, vascular disease, age 65–74 years, and sex; CHF, congestive heart failure; CPD, chronic pulmonary disease; DM, diabetes mellitus; HAS-BLED, hypertension, abnormal renal and liver function, stroke, bleeding, labile international normalized ratio, elderly, drugs, or alcohol; H2RA, H_2-receptor antagonist; HTN, hypertension; IS, ischemic stroke; IPTW, inverse probability of treatment weighting; MI, myocardial infarction; NHI, National Health Insurance; PPI, proton pump inhibitor; PSM, propensity score matching; PVD, peripheral vascular disease; RD, renal disease; R.Dabi, reduced dose of dabigatran.

Supplementary Table S10. Baseline characteristics for comparison 5 (standard dose of edoxaban vs warfarin)

Variables	Crude		PSM (logistic)		PSM (GBM)		IPTW (logistic)		IPTW (GBM)	
	S.Edo (N=10,971)	Warfarin (N=16,969)	S.Edo (N=9,647)	Warfarin (N=9,647)	S.Edo (N=9,154)	Warfarin (N=9,154)	S.Edo (N=10,971)	Warfarin (N=10,904)	S.Edo (N=10,971)	Warfarin (N=10,775)
	Mean/%	Mean/%	Mean/%	Mean/%	Mean/%	Mean/%	Mean/%	Mean/%	Mean/%	Mean/%
Age	68.17	67.16	68.01	68.08	68.03	68.16	68.17	68.20	68.17	68.18
CCI	3.21	3.49	3.28	3.32	3.37	3.35	3.21	3.26	3.21	3.22
CHA_2DS_2-VASc	3.96	3.88	3.95	3.98	4.02	4.03	3.96	3.99	3.96	3.96
HAS-BLED	3.34	3.28	3.32	3.34	3.35	3.36	3.34	3.35	3.34	3.34
Female	31.15	35.39	32.74	32.64	33.28	33.29	31.15	31.42	31.15	31.13
NHI	95.18	93.30	94.82	94.84	94.67	94.71	95.18	95.16	95.18	95.23
Medical aid	4.82	6.70	5.18	5.16	5.33	5.29	4.82	4.81	4.82	4.77
CHF	38.19	37.40	37.17	37.72	38.07	38.46	38.19	38.16	38.19	37.78
HTN	87.43	78.89	85.86	86.07	86.05	86.49	87.43	87.43	87.43	87.55
DM	44.58	42.66	43.88	44.57	45.55	45.85	44.58	45.08	44.58	44.57
IS	22.19	24.06	23.03	23.62	24.18	24.35	22.19	23.14	22.19	22.52
Bleeding	17.20	26.12	19.06	19.60	19.69	18.94	17.20	17.36	17.20	16.96
CPD	40.64	44.34	41.89	42.31	42.77	42.87	40.64	41.24	40.64	40.79
MI	4.08	6.82	4.55	4.55	4.78	4.62	4.08	4.24	4.08	4.18
PVD	24.14	24.05	24.51	24.39	25.05	24.80	24.14	24.10	24.14	23.98
RD	2.61	7.67	2.96	3.01	3.12	2.82	2.61	2.66	2.61	2.61
NSAIDs	99.25	98.56	99.16	99.14	99.22	99.19	99.25	99.27	99.25	99.31
Antiplatelets	82.56	82.26	82.89	82.56	82.82	83.46	82.56	82.49	82.56	82.51
Statins	71.72	67.96	71.29	71.69	72.45	72.47	71.72	72.38	71.72	72.10
Antiarrhythmics	68.99	73.99	71.16	70.97	71.71	71.65	68.99	69.28	68.99	69.22
PPI	81.92	81.31	81.94	82.14	82.19	82.27	81.92	82.01	81.92	81.88
H2RA	94.94	95.43	95.18	95.29	95.16	95.15	94.94	94.80	94.94	94.88
Digoxin	27.82	36.43	30.19	30.66	31.43	30.99	27.82	28.40	27.82	28.16

CCI, Charson Comorbidity Index; CHA_2DS_2-VASc, congestive heart failure, hypertension, age ≥75 years, diabetes mellitus, stroke, vascular disease, age 65–74 years, and sex; CHF, congestive heart failure; CPD, chronic pulmonary disease; DM, diabetes mellitus; HAS-BLED, hypertension, abnormal renal and liver function, stroke, bleeding, labile international normalized ratio, elderly, drugs, or alcohol; H2RA, H_2-receptor antagonist; HTN, hypertension; IS, ischemic stroke; IPTW, inverse probability of treatment weighting; MI, myocardial infarction; NHI, National Health Insurance; PPI, proton pump inhibitor; PSM, propensity score matching; PVD, peripheral vascular disease; RD, renal disease; S.Edo, standard dose of edoxaban.

Supplementary Table S11. Baseline characteristics for comparison 6 (reduced dose of edoxaban vs warfarin)

Variables	Crude		PSM (logistic)		PSM (GBM)		IPTW (logistic)		IPTW (GBM)	
	R.Edo (N=14,806)	Warfarin (N=16,969)	R.Edo (N=10,635)	Warfarin (N=10,635)	R.Edo (N=10,147)	Warfarin (N=10,147)	R.Edo (N=14,806)	Warfarin (N=14,783)	R.Edo (N=14,806)	Warfarin (N=14,479)
	Mean/%	Mean/%	Mean/%	Mean/%	Mean/%	Mean/%	Mean/%	Mean/%	Mean/%	Mean/%
Age	75.67	67.16	73.84	73.79	73.66	73.89	75.67	75.74	75.67	75.57
CCI	3.70	3.49	3.82	3.86	3.91	3.90	3.70	3.79	3.70	3.69
CHA_2DS_2-VASc	4.84	3.88	4.66	4.69	4.76	4.78	4.84	4.92	4.84	4.84
HAS-BLED	3.58	3.28	3.63	3.64	3.66	3.66	3.58	3.62	3.58	3.59
Female	52.73	35.39	46.12	45.99	47.43	47.99	52.73	53.99	52.73	52.51
NHI	91.82	93.30	92.47	92.20	91.87	92.19	91.82	91.71	91.82	91.92
Medical aid	8.18	6.70	7.53	7.80	8.13	7.81	8.18	8.29	8.18	8.08
CHF	43.74	37.40	41.02	41.08	41.60	41.39	43.74	44.22	43.74	43.17
HTN	87.80	78.89	85.76	86.06	87.53	87.43	87.80	88.69	87.80	87.91
DM	44.48	42.66	45.75	46.51	48.07	48.16	44.48	46.04	44.48	44.69
IS	22.79	24.06	25.95	26.93	27.55	27.64	22.79	24.02	22.79	23.03
Bleeding	21.72	26.12	25.62	25.88	25.87	25.40	21.72	22.12	21.72	21.61
CPD	47.83	44.34	48.73	49.24	49.44	48.89	47.83	48.25	47.83	48.06
MI	5.44	6.82	6.26	6.54	6.52	6.62	5.44	5.77	5.44	5.53
PVD	28.61	24.05	28.11	27.80	28.85	28.79	28.61	29.19	28.61	28.75
RD	6.13	7.67	7.75	8.10	8.00	7.83	6.13	6.60	6.13	6.16
NSAIDs	99.05	98.56	98.83	98.77	98.89	98.85	99.05	98.94	99.05	99.03
Antiplatelets	83.73	82.26	84.37	84.35	84.96	85.17	83.73	84.14	83.73	83.76
Statins	68.57	67.96	70.11	70.65	71.90	72.01	68.57	69.72	68.57	68.82
Antiarrhythmics	66.78	73.99	70.23	69.92	70.04	69.77	66.78	66.43	66.78	66.64
PPI	82.80	81.31	82.83	82.73	83.22	83.24	82.80	82.74	82.80	82.74
H2RA	96.12	95.43	96.37	96.47	96.21	96.15	96.12	96.05	96.12	96.07
Digoxin	37.18	36.43	38.85	39.20	39.71	40.07	37.18	38.22	37.18	37.65

CCI, Charson Comorbidity Index; CHA_2DS_2-VASc, congestive heart failure, hypertension, age ≥75 years, diabetes mellitus, stroke, vascular disease, age 65–74 years, and sex; CHF, congestive heart failure; CPD, chronic pulmonary disease; DM, diabetes mellitus; HAS-BLED, hypertension, abnormal renal and liver function, stroke, bleeding, labile international normalized ratio, elderly, drugs, or alcohol; H2RA, H_2-receptor antagonist; HTN, hypertension; IS, ischemic stroke; IPTW, inverse probability of treatment weighting; MI, myocardial infarction; NHI, National Health Insurance; PPI, proton pump inhibitor; PSM, propensity score matching; PVD, peripheral vascular disease; RD, renal disease; R.Edo, reduced dose of edoxaban.

Supplementary Table S12. Baseline characteristics for comparison 7 (standard dose of rivaroxaban vs warfarin)

Variables	Crude		PSM (logistic)		PSM (GBM)		IPTW (logistic)		IPTW (GBM)	
	S.Riva (N=17,631)	Warfarin (N=16,969)	S.Riva (N=13,324)	Warfarin (N=13,324)	S.Riva (N=12,443)	Warfarin (N=12,443)	S.Riva (N=17,631)	Warfarin (N=17,643)	S.Riva (N=17,631)	Warfarin (N=17,330)
	Mean/%	Mean/%	Mean/%	Mean/%	Mean/%	Mean/%	Mean/%	Mean/%	Mean/%	Mean/%
Age	69.98	67.16	69.24	69.39	69.62	69.80	69.98	70.24	69.98	70.00
CCI	3.51	3.49	3.52	3.52	3.63	3.63	3.51	3.59	3.51	3.50
CHA_2DS_2-VASc	4.31	3.88	4.18	4.18	4.30	4.33	4.31	4.39	4.31	4.32
HAS-BLED	3.47	3.28	3.41	3.41	3.47	3.48	3.47	3.51	3.47	3.48
Female	38.31	35.39	37.65	37.63	39.32	39.32	38.31	39.11	38.31	38.61
NHI	92.85	93.30	92.88	93.03	92.91	92.64	92.85	92.70	92.85	92.87
Medical aid	7.15	6.70	7.12	6.97	7.09	7.36	7.15	7.30	7.15	7.13
CHF	37.45	37.40	37.49	37.86	38.66	39.11	37.45	37.81	37.45	37.02
HTN	86.94	78.89	84.06	84.46	85.24	85.41	86.94	87.25	86.94	86.87
DM	47.69	42.66	45.90	45.64	47.79	48.01	47.69	48.92	47.69	47.62
IS	27.46	24.06	26.88	26.57	27.89	28.06	27.46	29.04	27.46	27.85
Bleeding	19.86	26.12	23.02	23.29	24.38	24.06	19.86	20.09	19.86	19.70
CPD	43.60	44.34	44.99	44.87	46.00	46.25	43.60	44.29	43.60	43.82
MI	4.43	6.82	5.46	5.52	5.78	5.59	4.43	4.60	4.43	4.52
PVD	26.26	24.05	25.59	25.66	26.75	26.39	26.26	26.44	26.26	25.97
RD	2.67	7.67	3.53	3.63	3.76	3.46	2.67	2.76	2.67	2.68
NSAIDs	99.11	98.56	98.92	98.93	98.90	98.98	99.11	99.09	99.11	99.12
Antiplatelets	84.79	82.26	83.74	83.89	84.52	84.59	84.79	84.83	84.79	84.70
Statins	71.81	67.96	70.79	70.50	71.75	72.03	71.81	72.56	71.81	71.96
Antiarrhythmics	66.63	73.99	70.94	71.01	71.50	70.93	66.63	66.31	66.63	66.75
PPI	81.95	81.31	81.74	81.85	81.91	82.34	81.95	82.09	81.95	82.01
H2RA	95.71	95.43	95.65	95.65	95.91	96.01	95.71	95.82	95.71	95.80
Digoxin	33.77	36.43	35.83	36.33	37.65	37.43	33.77	34.36	33.77	34.10

CCI, Charson Comorbidity Index; CHA_2DS_2-VASc, congestive heart failure, hypertension, age ≥75 years, diabetes mellitus, stroke, vascular disease, age 65–74 years, and sex; CHF, congestive heart failure; CPD, chronic pulmonary disease; DM, diabetes mellitus; HAS-BLED, hypertension, abnormal renal and liver function, stroke, bleeding, labile international normalized ratio, elderly, drugs, or alcohol; H2RA, H_2-receptor antagonist; HTN, hypertension; IS, ischemic stroke; IPTW, inverse probability of treatment weighting; MI, myocardial infarction; NHI, National Health Insurance; PPI, proton pump inhibitor; PSM, propensity score matching; PVD, peripheral vascular disease; RD, renal disease; S.Riva, standard dose of rivaroxaban.

Supplementary Table S13. Baseline characteristics for comparison 8 (reduced dose of rivaroxaban vs warfarin)

Variables	Crude		PSM (logistic)		PSM (GBM)		IPTW (logistic)		IPTW (GBM)	
	R.Riva (N=17,493)	Warfarin (N=16,969)	R.Riva (N=11,763)	Warfarin (N=11,763)	R.Riva (N=11,283)	Warfarin (N=11,283)	R.Riva (N=17,493)	Warfarin (N=17,488)	R.Riva (N=17,493)	Warfarin (N=17,188)
	Mean/%	Mean/%	Mean/%	Mean/%	Mean/%	Mean/%	Mean/%	Mean/%	Mean/%	Mean/%
Age	74.44	67.16	72.79	72.72	72.66	72.88	74.44	74.52	74.44	74.43
CCI	3.67	3.49	3.78	3.83	3.89	3.89	3.67	3.78	3.67	3.67
CHA_2DS_2-VASc	4.74	3.88	4.55	4.57	4.65	4.67	4.74	4.82	4.74	4.75
HAS-BLED	3.59	3.28	3.59	3.62	3.64	3.65	3.59	3.62	3.59	3.59
Female	46.49	35.39	42.54	42.29	43.20	43.45	46.49	47.37	46.49	46.71
NHI	92.44	93.30	92.73	92.37	92.48	92.31	92.44	92.28	92.44	92.38
Medical aid	7.56	6.70	7.27	7.63	7.52	7.69	7.56	7.72	7.56	7.62
CHF	42.22	37.40	39.42	39.94	41.23	41.03	42.22	42.70	42.22	41.59
HTN	89.12	78.89	85.77	86.32	87.61	87.89	89.12	89.84	89.12	89.19
DM	46.77	42.66	46.66	47.54	48.97	49.23	46.77	48.65	46.77	47.10
IS	23.51	24.06	26.66	27.04	28.070	28.15	23.51	24.67	23.51	23.75
Bleeding	21.00	26.12	25.27	26.18	25.68	25.81	21.00	21.19	21.00	20.86
CPD	47.03	44.34	48.08	48.30	48.96	49.04	47.03	47.90	47.03	47.38
MI	6.56	6.82	7.04	7.23	7.62	7.38	6.56	7.04	6.56	6.73
PVD	26.74	24.05	26.95	27.11	27.81	27.83	26.74	27.41	26.74	26.90
RD	5.00	7.67	6.95	7.52	7.29	7.13	5.00	5.25	5.00	4.96
NSAIDs	99.16	98.56	98.84	98.78	98.87	98.92	99.16	99.10	99.16	99.14
Antiplatelets	86.01	82.26	85.20	85.27	85.71	85.85	86.01	86.20	86.01	85.83
Statins	70.36	67.96	70.87	71.33	72.26	72.53	70.36	71.41	70.36	70.67
Antiarrhythmics	65.99	73.99	70.16	70.56	70.58	70.56	65.99	65.66	65.99	65.80
PPI	82.14	81.31	82.50	82.52	82.76	83.03	82.14	82.50	82.14	82.34
H2RA	96.41	95.43	96.10	96.33	96.35	96.35	96.41	96.38	96.41	96.28
Digoxin	35.83	36.43	37.96	38.60	39.31	39.48	35.83	36.71	35.83	36.27

CCI, Charson Comorbidity Index; CHA_2DS_2-VASc, congestive heart failure, hypertension, age ≥75 years, diabetes mellitus, stroke, vascular disease, age 65–74 years, and sex; CHF, congestive heart failure; CPD, chronic pulmonary disease; DM, diabetes mellitus; HAS-BLED, hypertension, abnormal renal and liver function, stroke, bleeding, labile international normalized ratio, elderly, drugs, or alcohol; H2RA, H_2-receptor antagonist; HTN, hypertension; IS, ischemic stroke; IPTW, inverse probability of treatment weighting; MI, myocardial infarction; NHI, National Health Insurance; PPI, proton pump inhibitor; PSM, propensity score matching; PVD, peripheral vascular disease; RD, renal disease; R.Riva, reduced dose of rivaroxaban.

Supplementary Table S14. Absolute standardized difference of baseline characteristics in comparison 1

Variables	Standard dose of Apixaban vs Warfarin				
	Crude	PSM (logistic)	PSM (GBM)	IPTW (logistic)	IPTW (GBM)
Age	0.117*	0.020	0.004	0.010	0.003
CCI	0.001*	0.000	0.002	0.033*	0.002
CHA_2DS_2-VASc	0.187*	0.006	0.006	0.040*	0.002
HAS-BLED	0.115*	0.001	0.002	0.034*	0.005
Female	0.044*	0.006	0.009	0.001	0.006
Insurance	0.057*	0.001	0.003	0.009	0.002
CHF	0.011	0.005	0.005	0.005	0.011
HTN	0.180*	0.010	0.007	0.017	0.004
DM	0.088*	0.006	0.010	0.028*	0.009
IS	0.177*	0.015	0.017	0.033*	0.006
Bleeding	0.147*	0.001	0.014	0.009	0.003
CPD	0.041*	0.012	0.002	0.014	0.001
MI	0.090*	0.008	0.004	0.004	0.002
PVD	0.013	0.004	0.003	0.006	0.000
RD	0.203*	0.006	0.018	0.002	0.002
NSAIDs	0.058*	0.002	0.008	0.001	0.005
Antiplatelets	0.006	0.006	0.009	0.009	0.006
Statins	0.159*	0.007	0.002	0.014	0.008
Antiarrhythmics	0.053*	0.004	0.007	0.003	0.005
PPI	0.048*	0.010	0.006	0.003	0.004
H2RA	0.012	0.004	0.010	0.002	0.001
Digoxin	0.182*	0.003	0.005	0.012	0.008

CCI, Charson Comorbidity Index; CHA_2DS_2-VASc, congestive heart failure, hypertension, age ≥75 years, diabetes mellitus, stroke, vascular disease, age 65–74 years, and sex; CHF, congestive heart failure; CPD, chronic pulmonary disease; DM, diabetes mellitus; HAS-BLED, hypertension, abnormal renal and liver function, stroke, bleeding, labile international normalized ratio, elderly, drugs, or alcohol; H2RA, H_2-receptor antagonist; HTN, hypertension; IS, ischemic stroke; IPTW, inverse probability of treatment weighting; MI, myocardial infarction; PPI, proton pump inhibitor; PSM, propensity score matching; PVD, peripheral vascular disease; RD, renal disease.

* *P*-value < 0.05.

Supplementary Table S15. Absolute standardized difference of baseline characteristics in comparison 2

Variables	Reduced dose of Apixaban vs Warfarin				
	**Crude**	**PSM (logistic)**	**PSM (GBM)**	**IPTW (logistic)**	**IPTW (GBM)**
Age	0.954*	0.018	0.013	0.014	0.010
CCI	0.271*	0.007	0.001	0.054*	0.004
CHA_2DS_2-VASc	0.714*	0.006	0.015	0.057*	0.001
HAS-BLED	0.420*	0.020	0.012	0.037*	0.003
Female	0.390*	0.016	0.007	0.026*	0.002
Insurance	0.063*	0.002	0.000	0.013	0.003
CHF	0.153*	0.010	0.006	0.019	0.009
HTN	0.272*	0.005	0.009	0.038*	0.003
DM	0.130*	0.004	0.001	0.040*	0.001
IS	0.174*	0.004	0.013	0.037*	0.003
Bleeding	0.027*	0.008	0.008	0.019	0.003
CPD	0.128*	0.006	0.005	0.011	0.001
MI	0.041*	0.006	0.005	0.027*	0.011
PVD	0.100*	0.010	0.002	0.004	0.001
RD	0.031*	0.005	0.002	0.026*	0.000
NSAIDs	0.027*	0.014	0.002	0.014	0.000
Antiplatelets	0.071*	0.011	0.000	0.020	0.005
Statins	0.083*	0.011	0.008	0.033*	0.010
Antiarrhythmics	0.073*	0.002	0.004	0.008	0.000
PPI	0.072*	0.004	0.005	0.010	0.008
H2RA	0.058*	0.012	0.011	0.004	0.001
Digoxin	0.057*	0.001	0.014	0.015	0.007

CCI, Charson Comorbidity Index; CHA_2DS_2-VASc, congestive heart failure, hypertension, age ≥75 years, diabetes mellitus, stroke, vascular disease, age 65–74 years, and sex; CHF, congestive heart failure; CPD, chronic pulmonary disease; DM, diabetes mellitus; HAS-BLED, hypertension, abnormal renal and liver function, stroke, bleeding, labile international normalized ratio, elderly, drugs, or alcohol; H2RA, H_2-receptor antagonist; HTN, hypertension; IS, ischemic stroke; IPTW, inverse probability of treatment weighting; MI, myocardial infarction; PPI, proton pump inhibitor; PSM, propensity score matching; PVD, peripheral vascular disease; RD, renal disease.

* *P*-value < 0.05.

Supplementary Table S16. Absolute standardized difference of baseline characteristics in comparison 3

Variables	Standard dose of Dabigatran vs Warfarin				
	Crude	PSM (logistic)	PSM (GBM)	IPTW (logistic)	IPTW (GBM)
Age	0.050*	0.036*	0.007	0.026	0.008
CCI	0.029*	0.025	0.013	0.028	0.003
CHA_2DS_2-VASc	0.124*	0.040*	0.002	0.043*	0.003
HAS-BLED	0.080*	0.032	0.001	0.036*	0.002
Female	0.066*	0.010	0.019	0.008	0.000
Insurance	0.045*	0.001	0.009	0.010	0.003
CHF	0.020	0.018	0.003	0.001	0.011
HTN	0.158*	0.028	0.029	0.018	0.009
DM	0.116*	0.016	0.003	0.018	0.004
IS	0.270*	0.001	0.011	0.021	0.001
Bleeding	0.217*	0.002	0.016	0.004	0.005
CPD	0.088*	0.021	0.006	0.008	0.002
MI	0.155*	0.000	0.027	0.002	0.003
PVD	0.015	0.024	0.002	0.005	0.004
RD	0.259*	0.009	0.028	0.003	0.002
NSAIDs	0.038*	0.007	0.003	0.002	0.003
Antiplatelets	0.091*	0.013	0.015	0.010	0.010
Statins	0.217*	0.017	0.025	0.003	0.003
Antiarrhythmics	0.023	0.004	0.008	0.003	0.003
PPI	0.041*	0.003	0.005	0.005	0.003
H2RA	0.006	0.010	0.008	0.000	0.003
Digoxin	0.145*	0.001	0.008	0.006	0.007

CCI, Charson Comorbidity Index; CHA_2DS_2-VASc, congestive heart failure, hypertension, age ≥75 years, diabetes mellitus, stroke, vascular disease, age 65–74 years, and sex; CHF, congestive heart failure; CPD, chronic pulmonary disease; DM, diabetes mellitus; HAS-BLED, hypertension, abnormal renal and liver function, stroke, bleeding, labile international normalized ratio, elderly, drugs, or alcohol; H2RA, H_2-receptor antagonist; HTN, hypertension; IS, ischemic stroke; IPTW, inverse probability of treatment weighting; MI, myocardial infarction; PPI, proton pump inhibitor; PSM, propensity score matching; PVD, peripheral vascular disease; RD, renal disease.

* *P*-value < 0.05.

Supplementary Table S17. Absolute standardized difference of baseline characteristics in comparison 4

Variables	Reduced dose of Dabigatran vs Warfarin				
	**Crude**	**PSM (logistic)**	**PSM (GBM)**	**IPTW (logistic)**	**IPTW (GBM)**
Age	0.641*	0.005	0.029*	0.007	0.004
CCI	0.102*	0.025	0.012	0.037*	0.002
CHA_2DS_2-VASc	0.516*	0.007	0.022	0.046*	0.005
HAS-BLED	0.317*	0.031*	0.028*	0.031*	0.004
Female	0.238*	0.006	0.001	0.018	0.001
Insurance	0.058*	0.008	0.003	0.007	0.002
CHF	0.041*	0.005	0.003	0.003	0.011
HTN	0.283*	0.012	0.009	0.018	0.000
DM	0.103*	0.021	0.012	0.033*	0.011
IS	0.153*	0.006	0.012	0.038*	0.009
Bleeding	0.109*	0.029*	0.017	0.008	0.001
CPD	0.037*	0.020	0.016	0.006	0.002
MI	0.063*	0.006	0.008	0.007	0.005
PVD	0.080*	0.001	0.007	0.006	0.004
RD	0.169*	0.013	0.007	0.004	0.001
NSAIDs	0.035*	0.004	0.002	0.008	0.006
Antiplatelets	0.117*	0.009	0.015	0.004	0.001
Statins	0.140*	0.000	0.008	0.024	0.006
Antiarrhythmics	0.077*	0.001	0.000	0.008	0.006
PPI	0.082*	0.002	0.006	0.008	0.003
H2RA	0.077*	0.005	0.005	0.003	0.004
Digoxin	0.013	0.015	0.013	0.006	0.003

CCI, Charson Comorbidity Index; CHA_2DS_2-VASc, congestive heart failure, hypertension, age ≥75 years, diabetes mellitus, stroke, vascular disease, age 65–74 years, and sex; CHF, congestive heart failure; CPD, chronic pulmonary disease; DM, diabetes mellitus; HAS-BLED, hypertension, abnormal renal and liver function, stroke, bleeding, labile international normalized ratio, elderly, drugs, or alcohol; H2RA, H_2-receptor antagonist; HTN, hypertension; IS, ischemic stroke; IPTW, inverse probability of treatment weighting; MI, myocardial infarction; PPI, proton pump inhibitor; PSM, propensity score matching; PVD, peripheral vascular disease; RD, renal disease.

* *P*-value < 0.05.

Supplementary Table S18. Absolute standardized difference of baseline characteristics in comparison 5

Variables	Standard dose of Edoxaban vs Warfarin				
	**Crude**	**PSM (logistic)**	**PSM (GBM)**	**IPTW (logistic)**	**IPTW (GBM)**
Age	0.088*	0.007	0.012	0.004	0.002
CCI	0.118*	0.018	0.008	0.023	0.002
CHA_2DS_2-VASc	0.043*	0.019	0.008	0.018	0.001
HAS-BLED	0.043*	0.019	0.008	0.018	0.004
Female	0.090*	0.002	0.000	0.006	0.000
Insurance	0.081*	0.001	0.002	0.001	0.002
CHF	0.016	0.011	0.008	0.001	0.009
HTN	0.230*	0.006	0.013	0.000	0.003
DM	0.039*	0.014	0.006	0.010	0.000
IS	0.044*	0.014	0.004	0.023	0.008
Bleeding	0.218*	0.014	0.019	0.004	0.006
CPD	0.075*	0.009	0.002	0.012	0.003
MI	0.121*	0.000	0.008	0.008	0.005
PVD	0.002	0.003	0.006	0.001	0.004
RD	0.231*	0.002	0.018	0.003	0.000
NSAIDs	0.066*	0.002	0.004	0.003	0.006
Antiplatelets	0.008	0.009	0.017	0.002	0.002
Statins	0.082*	0.009	0.000	0.015	0.009
Antiarrhythmics	0.111*	0.004	0.001	0.006	0.005
PPI	0.016	0.005	0.002	0.003	0.001
H2RA	0.023	0.005	0.001	0.006	0.003
Digoxin	0.185*	0.010	0.009	0.013	0.008

CCI, Charson Comorbidity Index; CHA_2DS_2-VASc, congestive heart failure, hypertension, age ≥75 years, diabetes mellitus, stroke, vascular disease, age 65–74 years, and sex; CHF, congestive heart failure; CPD, chronic pulmonary disease; DM, diabetes mellitus; HAS-BLED, hypertension, abnormal renal and liver function, stroke, bleeding, labile international normalized ratio, elderly, drugs, or alcohol; H2RA, H_2-receptor antagonist; HTN, hypertension; IS, ischemic stroke; IPTW, inverse probability of treatment weighting; MI, myocardial infarction; PPI, proton pump inhibitor; PSM, propensity score matching; PVD, peripheral vascular disease; RD, renal disease.

* *P*-value < 0.05.

Supplementary Table S19. Absolute standardized difference of baseline characteristics in comparison 6

Variables	Reduced dose of Edoxaban vs Warfarin				
	Crude	PSM (logistic)	PSM (GBM)	IPTW (logistic)	IPTW (GBM)
Age	0.757*	0.006	0.024	0.009	0.011
CCI	0.084*	0.017	0.005	0.037*	0.004
CHA_2DS_2-VASc	0.504*	0.013	0.011	0.046*	0.003
HAS-BLED	0.254*	0.017	0.001	0.030*	0.001
Female	0.355*	0.003	0.011	0.025*	0.004
Insurance	0.056*	0.010	0.012	0.004	0.004
CHF	0.129*	0.001	0.004	0.010	0.011
HTN	0.241*	0.008	0.003	0.028*	0.004
DM	0.037*	0.015	0.002	0.031*	0.004
IS	0.030*	0.022	0.002	0.029*	0.006
Bleeding	0.103*	0.006	0.011	0.010	0.003
CPD	0.070*	0.010	0.011	0.008	0.005
MI	0.058*	0.011	0.004	0.014	0.004
PVD	0.104*	0.007	0.001	0.013	0.003
RD	0.061*	0.013	0.006	0.019	0.002
NSAIDs	0.045*	0.006	0.004	0.011	0.002
Antiplatelets	0.039*	0.001	0.006	0.011	0.001
Statins	0.013	0.012	0.002	0.025*	0.005
Antiarrhythmics	0.158*	0.007	0.006	0.007	0.003
PPI	0.039*	0.003	0.001	0.002	0.002
H2RA	0.035*	0.006	0.003	0.004	0.003
Digoxin	0.016	0.007	0.007	0.021	0.010

CCI, Charson Comorbidity Index; CHA_2DS_2-VASc, congestive heart failure, hypertension, age ≥75 years, diabetes mellitus, stroke, vascular disease, age 65–74 years, and sex; CHF, congestive heart failure; CPD, chronic pulmonary disease; DM, diabetes mellitus; HAS-BLED, hypertension, abnormal renal and liver function, stroke, bleeding, labile international normalized ratio, elderly, drugs, or alcohol; H2RA, H_2-receptor antagonist; HTN, hypertension; IS, ischemic stroke; IPTW, inverse probability of treatment weighting; MI, myocardial infarction; PPI, proton pump inhibitor; PSM, propensity score matching; PVD, peripheral vascular disease; RD, renal disease.

* *P*-value < 0.05.

Supplementary Table S20. Absolute standardized difference of baseline characteristics in comparison 7

Variables	Standard dose of Rivaroxaban vs Warfarin				
	**Crude**	**PSM (logistic)**	**PSM (GBM)**	**IPTW (logistic)**	**IPTW (GBM)**
Age	0.241*	0.014	0.016	0.025*	0.002
CCI	0.006	0.001	0.001	0.032*	0.005
CHA_2DS_2-VASc	0.222*	0.002	0.013	0.043*	0.002
HAS-BLED	0.157*	0.005	0.010	0.030*	0.002
Female	0.061*	0.000	0.000	0.016	0.006
Insurance	0.018	0.006	0.011	0.006	0.001
CHF	0.001	0.008	0.009	0.007	0.009
HTN	0.215*	0.011	0.005	0.009	0.002
DM	0.101*	0.005	0.005	0.025*	0.001
IS	0.078*	0.007	0.004	0.035*	0.009
Bleeding	0.149*	0.006	0.007	0.006	0.004
CPD	0.015	0.002	0.005	0.014	0.004
MI	0.104*	0.003	0.008	0.008	0.005
PVD	0.051*	0.002	0.008	0.004	0.007
RD	0.227*	0.005	0.015	0.005	0.000
NSAIDs	0.051*	0.001	0.008	0.003	0.001
Antiplatelets	0.068*	0.004	0.002	0.001	0.003
Statins	0.084*	0.006	0.006	0.017	0.003
Antiarrhythmics	0.162*	0.001	0.013	0.007	0.003
PPI	0.017	0.003	0.011	0.003	0.002
H2RA	0.014	0.000	0.005	0.006	0.004
Digoxin	0.056*	0.010	0.005	0.012	0.007

CCI, Charson Comorbidity Index; CHA_2DS_2-VASc, congestive heart failure, hypertension, age ≥75 years, diabetes mellitus, stroke, vascular disease, age 65–74 years, and sex; CHF, congestive heart failure; CPD, chronic pulmonary disease; DM, diabetes mellitus; HAS-BLED, hypertension, abnormal renal and liver function, stroke, bleeding, labile international normalized ratio, elderly, drugs, or alcohol; H2RA, H_2-receptor antagonist; HTN, hypertension; IS, ischemic stroke; IPTW, inverse probability of treatment weighting; MI, myocardial infarction; PPI, proton pump inhibitor; PSM, propensity score matching; PVD, peripheral vascular disease; RD, renal disease.

* *P*-value < 0.05.

Supplementary Table S21. Absolute standardized difference of baseline characteristics in comparison 8

Variables	Reduced dose of Rivaroxaban vs Warfarin				
	Crude	PSM (logistic)	PSM (GBM)	IPTW (logistic)	IPTW (GBM)
Age	0.650*	0.007	0.023	0.009	0.001
CCI	0.073*	0.021	0.002	0.041*	0.001
CHA_2DS_2-VASc	0.447*	0.012	0.012	0.044*	0.002
HAS-BLED	0.259*	0.027*	0.013	0.029*	0.001
Female	0.227*	0.005	0.005	0.018	0.004
Insurance	0.034*	0.014	0.006	0.006	0.002
CHF	0.099*	0.011	0.004	0.010	0.013
HTN	0.282*	0.016	0.009	0.023*	0.002
DM	0.083*	0.018	0.005	0.038*	0.007
IS	0.013	0.009	0.002	0.027*	0.006
Bleeding	0.121*	0.021	0.003	0.005	0.003
CPD	0.054*	0.004	0.002	0.017	0.007
MI	0.010	0.007	0.009	0.019	0.007
PVD	0.062*	0.004	0.000	0.015	0.004
RD	0.110*	0.022	0.006	0.012	0.002
NSAIDs	0.056*	0.005	0.005	0.006	0.002
Antiplatelets	0.103*	0.002	0.004	0.006	0.005
Statins	0.052*	0.010	0.006	0.023*	0.007
Antiarrhythmics	0.175*	0.009	0.001	0.007	0.004
PPI	0.021*	0.001	0.007	0.010	0.005
H2RA	0.050*	0.012	0.000	0.002	0.007
Digoxin	0.013	0.013	0.004	0.018	0.009

CCI, Charson Comorbidity Index; CHA_2DS_2-VASc, congestive heart failure, hypertension, age ≥75 years, diabetes mellitus, stroke, vascular disease, age 65–74 years, and sex; CHF, congestive heart failure; CPD, chronic pulmonary disease; DM, diabetes mellitus; HAS-BLED, hypertension, abnormal renal and liver function, stroke, bleeding, labile international normalized ratio, elderly, drugs, or alcohol; H2RA, H_2-receptor antagonist; HTN, hypertension; IS, ischemic stroke; IPTW, inverse probability of treatment weighting; MI, myocardial infarction; PPI, proton pump inhibitor; PSM, propensity score matching; PVD, peripheral vascular disease; RD, renal disease.

* *P*-value < 0.05.

Variables | Standard dose of Apixaban vs Warfarin

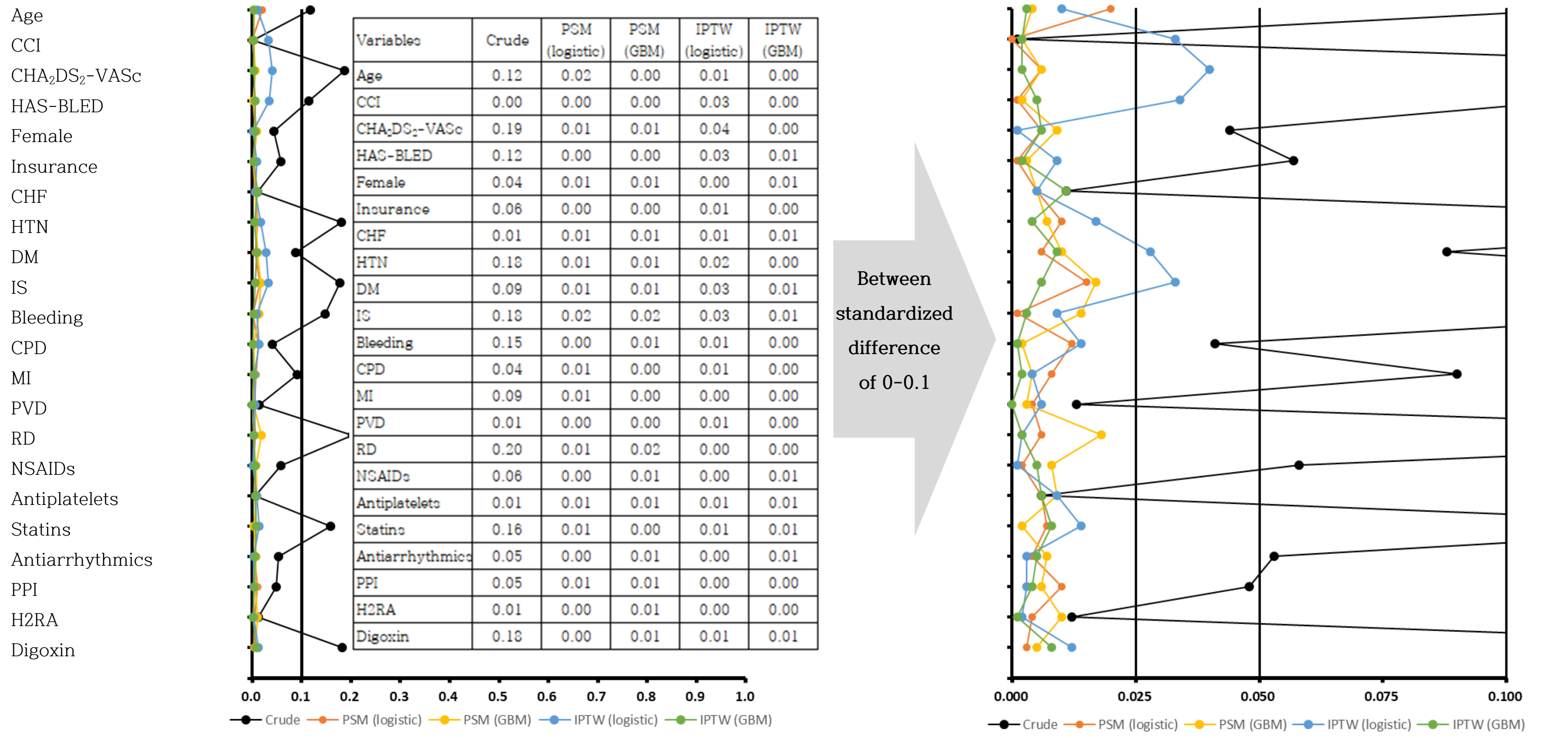

Variables	Crude	PSM (logistic)	PSM (GBM)	IPTW (logistic)	IPTW (GBM)
Age	0.12	0.02	0.00	0.01	0.00
CCI	0.00	0.00	0.00	0.03	0.00
CHA_2DS_2-VASc	0.19	0.01	0.01	0.04	0.00
HAS-BLED	0.12	0.00	0.00	0.03	0.01
Female	0.04	0.01	0.01	0.00	0.01
Insurance	0.06	0.00	0.00	0.01	0.00
CHF	0.01	0.01	0.01	0.01	0.01
HTN	0.18	0.01	0.01	0.02	0.00
DM	0.09	0.01	0.01	0.03	0.01
IS	0.18	0.02	0.02	0.03	0.01
Bleeding	0.15	0.00	0.01	0.01	0.00
CPD	0.04	0.01	0.00	0.01	0.00
MI	0.09	0.01	0.00	0.00	0.00
PVD	0.01	0.00	0.00	0.01	0.00
RD	0.20	0.01	0.02	0.00	0.00
NSAIDs	0.06	0.00	0.01	0.00	0.01
Antiplatelets	0.01	0.01	0.01	0.01	0.01
Statins	0.16	0.01	0.00	0.01	0.01
Antiarrhythmics	0.05	0.00	0.01	0.00	0.01
PPI	0.05	0.01	0.01	0.00	0.00
H2RA	0.01	0.00	0.01	0.00	0.00
Digoxin	0.18	0.00	0.01	0.01	0.01

CCI, Charson Comorbidity Index; CHA2DS2-VASc, congestive heart failure, hypertension, age ≥75 years, diabetes mellitus, stroke, vascular disease, age 65–74 years, and sex; CHF, congestive heart failure; CPD, chronic pulmonary disease; DM, diabetes mellitus; HAS-BLED, hypertension, abnormal renal and liver function, stroke, bleeding, labile international normalized ratio, elderly, drugs, or alcohol; H2RA, H2-receptor antagonist; HTN, hypertension; IS, ischemic stroke; IPTW, inverse probability of treatment weighting; MI, myocardial infarction; PPI, proton pump inhibitor; PSM, propensity score matching; PVD, peripheral vascular disease; RD, renal disease. Conventional approaches are PSM (logistic) and IPTW (logistic) and machine learning approaches are PSM (GBM) and IPTW (GBM).

Supplementary Figure S1. Absolute standardized differences in comparison 1

Variables | Reduced dose of Apixaban vs Warfarin

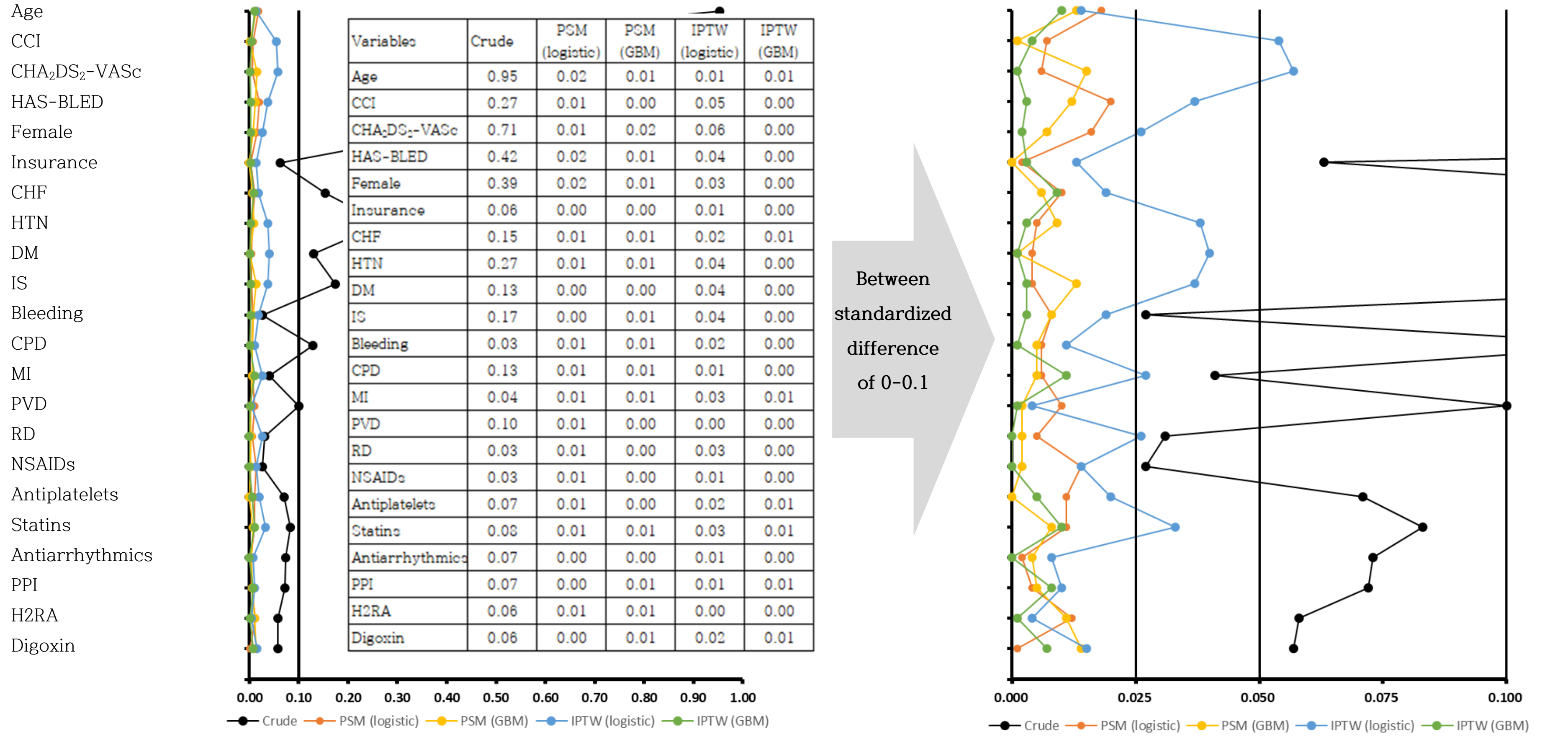

Variables	Crude	PSM (logistic)	PSM (GBM)	IPTW (logistic)	IPTW (GBM)
Age	0.95	0.02	0.01	0.01	0.01
CCI	0.27	0.01	0.00	0.05	0.00
CHA_2DS_2-VASc	0.71	0.01	0.02	0.06	0.00
HAS-BLED	0.42	0.02	0.01	0.04	0.00
Female	0.39	0.02	0.01	0.03	0.00
Insurance	0.06	0.00	0.00	0.01	0.00
CHF	0.15	0.01	0.01	0.02	0.01
HTN	0.27	0.01	0.01	0.04	0.00
DM	0.13	0.00	0.00	0.04	0.00
IS	0.17	0.00	0.01	0.04	0.00
Bleeding	0.03	0.01	0.01	0.02	0.00
CPD	0.13	0.01	0.01	0.01	0.00
MI	0.04	0.01	0.01	0.03	0.01
PVD	0.10	0.01	0.00	0.00	0.00
RD	0.03	0.01	0.00	0.03	0.00
NSAIDs	0.03	0.01	0.00	0.01	0.00
Antiplatelets	0.07	0.01	0.00	0.02	0.01
Statins	0.08	0.01	0.01	0.03	0.01
Antiarrhythmics	0.07	0.00	0.00	0.01	0.00
PPI	0.07	0.00	0.01	0.01	0.01
H2RA	0.06	0.01	0.01	0.00	0.00
Digoxin	0.06	0.00	0.01	0.02	0.01

CCI, Charson Comorbidity Index; CHA2DS2-VASc, congestive heart failure, hypertension, age ≥75 years, diabetes mellitus, stroke, vascular disease, age 65–74 years, and sex; CHF, congestive heart failure; CPD, chronic pulmonary disease; DM, diabetes mellitus; HAS-BLED, hypertension, abnormal renal and liver function, stroke, bleeding, labile international normalized ratio, elderly, drugs, or alcohol; H2RA, H2-receptor antagonist; HTN, hypertension; IS, ischemic stroke; IPTW, inverse probability of treatment weighting; MI, myocardial infarction; PPI, proton pump inhibitor; PSM, propensity score matching; PVD, peripheral vascular disease; RD, renal disease. Conventional approaches are PSM (logistic) and IPTW (logistic) and machine learning approaches are PSM (GBM) and IPTW (GBM).

Supplementary Figure S2. Absolute standardized differences in comparison 2

Variables	Standard dose of Dabigatran vs Warfarin

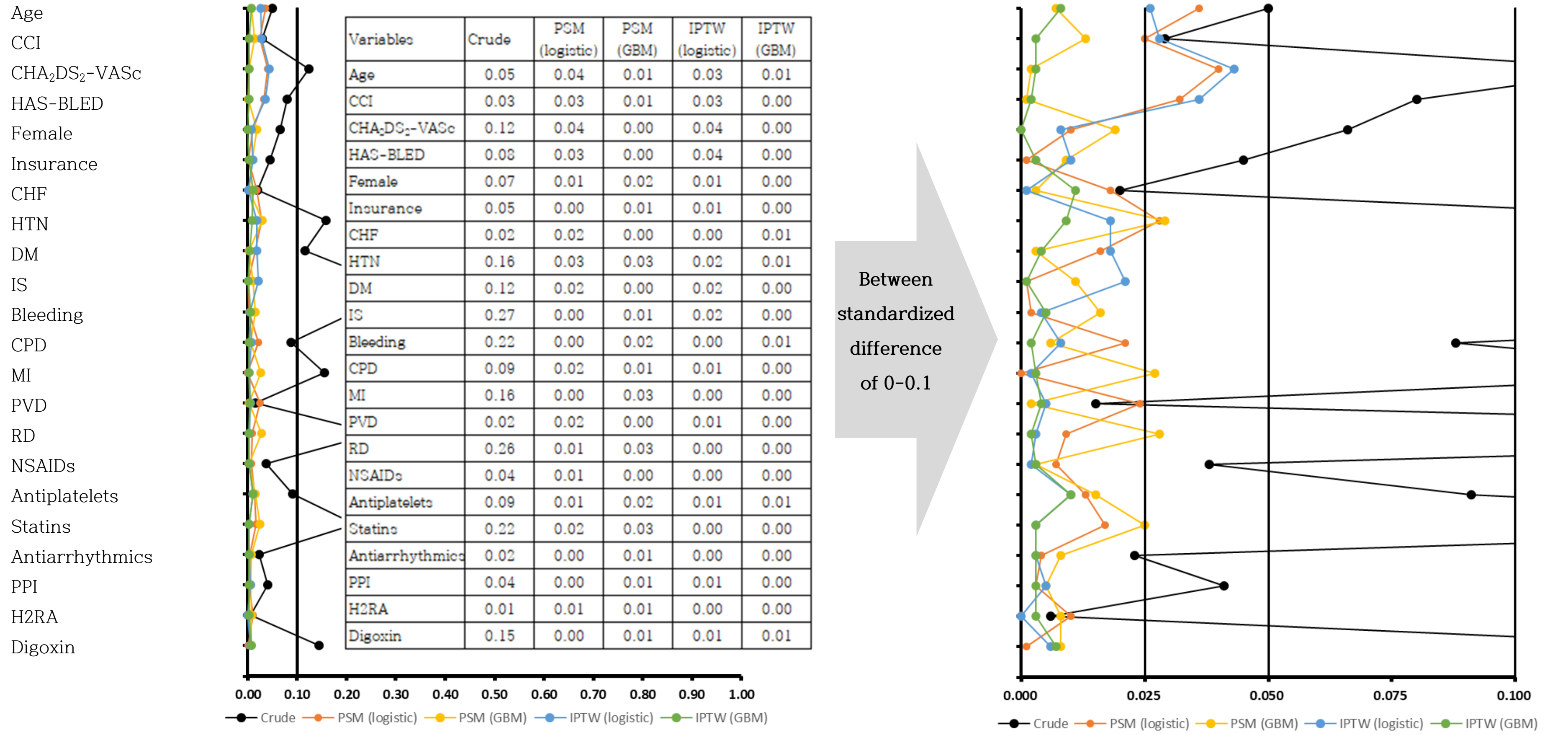

Variables	Crude	PSM (logistic)	PSM (GBM)	IPTW (logistic)	IPTW (GBM)
Age	0.05	0.04	0.01	0.03	0.01
CCI	0.03	0.03	0.01	0.03	0.00
CHA_2DS_2-VASc	0.12	0.04	0.00	0.04	0.00
HAS-BLED	0.08	0.03	0.00	0.04	0.00
Female	0.07	0.01	0.02	0.01	0.00
Insurance	0.05	0.00	0.01	0.01	0.00
CHF	0.02	0.02	0.00	0.00	0.01
HTN	0.16	0.03	0.03	0.02	0.01
DM	0.12	0.02	0.00	0.02	0.00
IS	0.27	0.00	0.01	0.02	0.00
Bleeding	0.22	0.00	0.02	0.00	0.01
CPD	0.09	0.02	0.01	0.01	0.00
MI	0.16	0.00	0.03	0.00	0.00
PVD	0.02	0.02	0.00	0.01	0.00
RD	0.26	0.01	0.03	0.00	0.00
NSAIDs	0.04	0.01	0.00	0.00	0.00
Antiplatelets	0.09	0.01	0.02	0.01	0.01
Statins	0.22	0.02	0.03	0.00	0.00
Antiarrhythmics	0.02	0.00	0.01	0.00	0.00
PPI	0.04	0.00	0.01	0.01	0.00
H2RA	0.01	0.01	0.01	0.00	0.00
Digoxin	0.15	0.00	0.01	0.01	0.01

CCI, Charson Comorbidity Index; CHA2DS2-VASc, congestive heart failure, hypertension, age ≥75 years, diabetes mellitus, stroke, vascular disease, age 65–74 years, and sex; CHF, congestive heart failure; CPD, chronic pulmonary disease; DM, diabetes mellitus; HAS-BLED, hypertension, abnormal renal and liver function, stroke, bleeding, labile international normalized ratio, elderly, drugs, or alcohol; H2RA, H2-receptor antagonist; HTN, hypertension; IS, ischemic stroke; IPTW, inverse probability of treatment weighting; MI, myocardial infarction; PPI, proton pump inhibitor; PSM, propensity score matching; PVD, peripheral vascular disease; RD, renal disease. Conventional approaches are PSM (logistic) and IPTW (logistic) and machine learning approaches are PSM (GBM) and IPTW (GBM).

Supplementary Figure S3. Absolute standardized differences in comparison 3

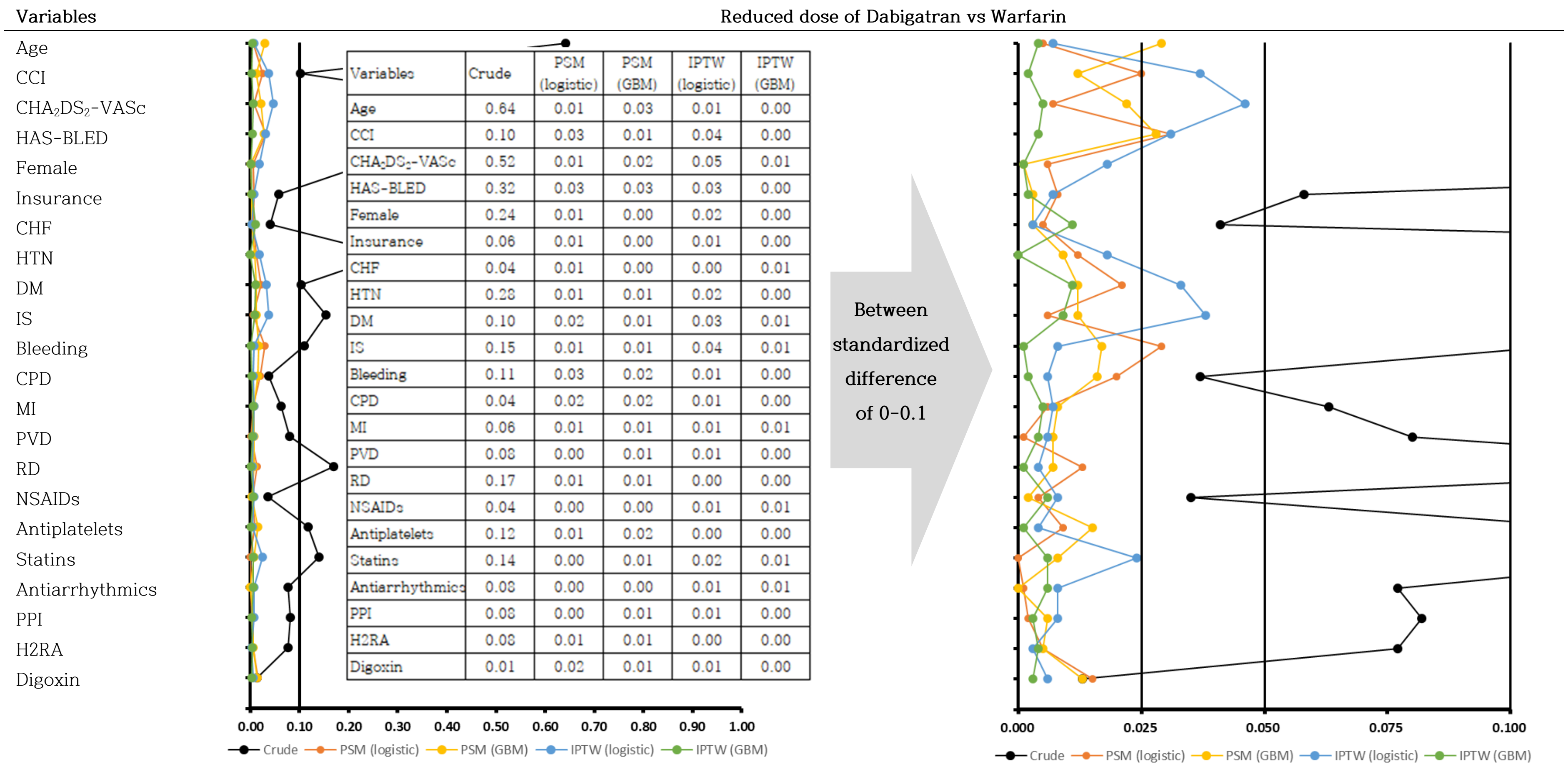

Variables	Crude	PSM (logistic)	PSM (GBM)	IPTW (logistic)	IPTW (GBM)
Age	0.64	0.01	0.03	0.01	0.00
CCI	0.10	0.03	0.01	0.04	0.00
CHA_2DS_2-VASc	0.52	0.01	0.02	0.05	0.01
HAS-BLED	0.32	0.03	0.03	0.03	0.00
Female	0.24	0.01	0.00	0.02	0.00
Insurance	0.06	0.01	0.00	0.01	0.00
CHF	0.04	0.01	0.00	0.00	0.01
HTN	0.28	0.01	0.01	0.02	0.00
DM	0.10	0.02	0.01	0.03	0.01
IS	0.15	0.01	0.01	0.04	0.01
Bleeding	0.11	0.03	0.02	0.01	0.00
CPD	0.04	0.02	0.02	0.01	0.00
MI	0.06	0.01	0.01	0.01	0.01
PVD	0.08	0.00	0.01	0.01	0.00
RD	0.17	0.01	0.01	0.00	0.00
NSAIDs	0.04	0.00	0.00	0.01	0.01
Antiplatelets	0.12	0.01	0.02	0.00	0.00
Statins	0.14	0.00	0.01	0.02	0.01
Antiarrhythmics	0.08	0.00	0.00	0.01	0.01
PPI	0.08	0.00	0.01	0.01	0.00
H2RA	0.08	0.01	0.01	0.00	0.00
Digoxin	0.01	0.02	0.01	0.01	0.00

CCI, Charson Comorbidity Index; CHA2DS2-VASc, congestive heart failure, hypertension, age ≥75 years, diabetes mellitus, stroke, vascular disease, age 65–74 years, and sex; CHF, congestive heart failure; CPD, chronic pulmonary disease; DM, diabetes mellitus; HAS-BLED, hypertension, abnormal renal and liver function, stroke, bleeding, labile international normalized ratio, elderly, drugs, or alcohol; H2RA, H2-receptor antagonist; HTN, hypertension; IS, ischemic stroke; IPTW, inverse probability of treatment weighting; MI, myocardial infarction; PPI, proton pump inhibitor; PSM, propensity score matching; PVD, peripheral vascular disease; RD, renal disease. Conventional approaches are PSM (logistic) and IPTW (logistic) and machine learning approaches are PSM (GBM) and IPTW (GBM).

Supplementary Figure S4. Absolute standardized differences in comparison 4

Variables	Standard dose of Edoxaban vs Warfarin

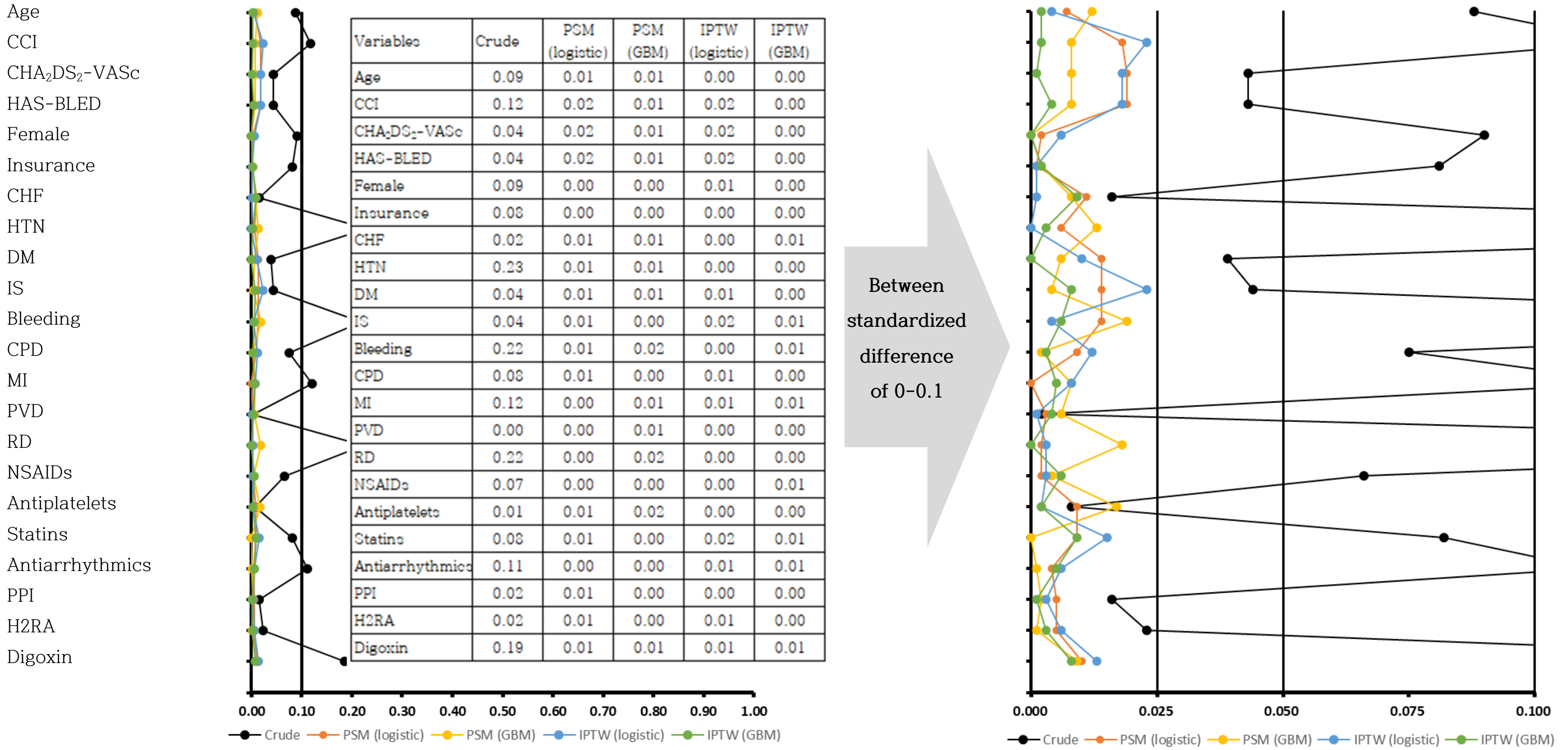

Variables	Crude	PSM (logistic)	PSM (GBM)	IPTW (logistic)	IPTW (GBM)
Age	0.09	0.01	0.01	0.00	0.00
CCI	0.12	0.02	0.01	0.02	0.00
CHA_2DS_2-VASc	0.04	0.02	0.01	0.02	0.00
HAS-BLED	0.04	0.02	0.01	0.02	0.00
Female	0.09	0.00	0.00	0.01	0.00
Insurance	0.08	0.00	0.00	0.00	0.00
CHF	0.02	0.01	0.01	0.00	0.01
HTN	0.23	0.01	0.01	0.00	0.00
DM	0.04	0.01	0.01	0.01	0.00
IS	0.04	0.01	0.00	0.02	0.01
Bleeding	0.22	0.01	0.02	0.00	0.01
CPD	0.08	0.01	0.00	0.01	0.00
MI	0.12	0.00	0.01	0.01	0.01
PVD	0.00	0.00	0.01	0.00	0.00
RD	0.22	0.00	0.02	0.00	0.00
NSAIDs	0.07	0.00	0.00	0.00	0.01
Antiplatelets	0.01	0.01	0.02	0.00	0.00
Statins	0.08	0.01	0.00	0.02	0.01
Antiarrhythmics	0.11	0.00	0.00	0.01	0.01
PPI	0.02	0.01	0.00	0.00	0.00
H2RA	0.02	0.01	0.00	0.01	0.00
Digoxin	0.19	0.01	0.01	0.01	0.01

CCI, Charson Comorbidity Index; CHA2DS2-VASc, congestive heart failure, hypertension, age ≥75 years, diabetes mellitus, stroke, vascular disease, age 65–74 years, and sex; CHF, congestive heart failure; CPD, chronic pulmonary disease; DM, diabetes mellitus; HAS-BLED, hypertension, abnormal renal and liver function, stroke, bleeding, labile international normalized ratio, elderly, drugs, or alcohol; H2RA, H2-receptor antagonist; HTN, hypertension; IS, ischemic stroke; IPTW, inverse probability of treatment weighting; MI, myocardial infarction; PPI, proton pump inhibitor; PSM, propensity score matching; PVD, peripheral vascular disease; RD, renal disease. Conventional approaches are PSM (logistic) and IPTW (logistic) and machine learning approaches are PSM (GBM) and IPTW (GBM).

Supplementary Figure S5. Absolute standardized differences in comparison 5

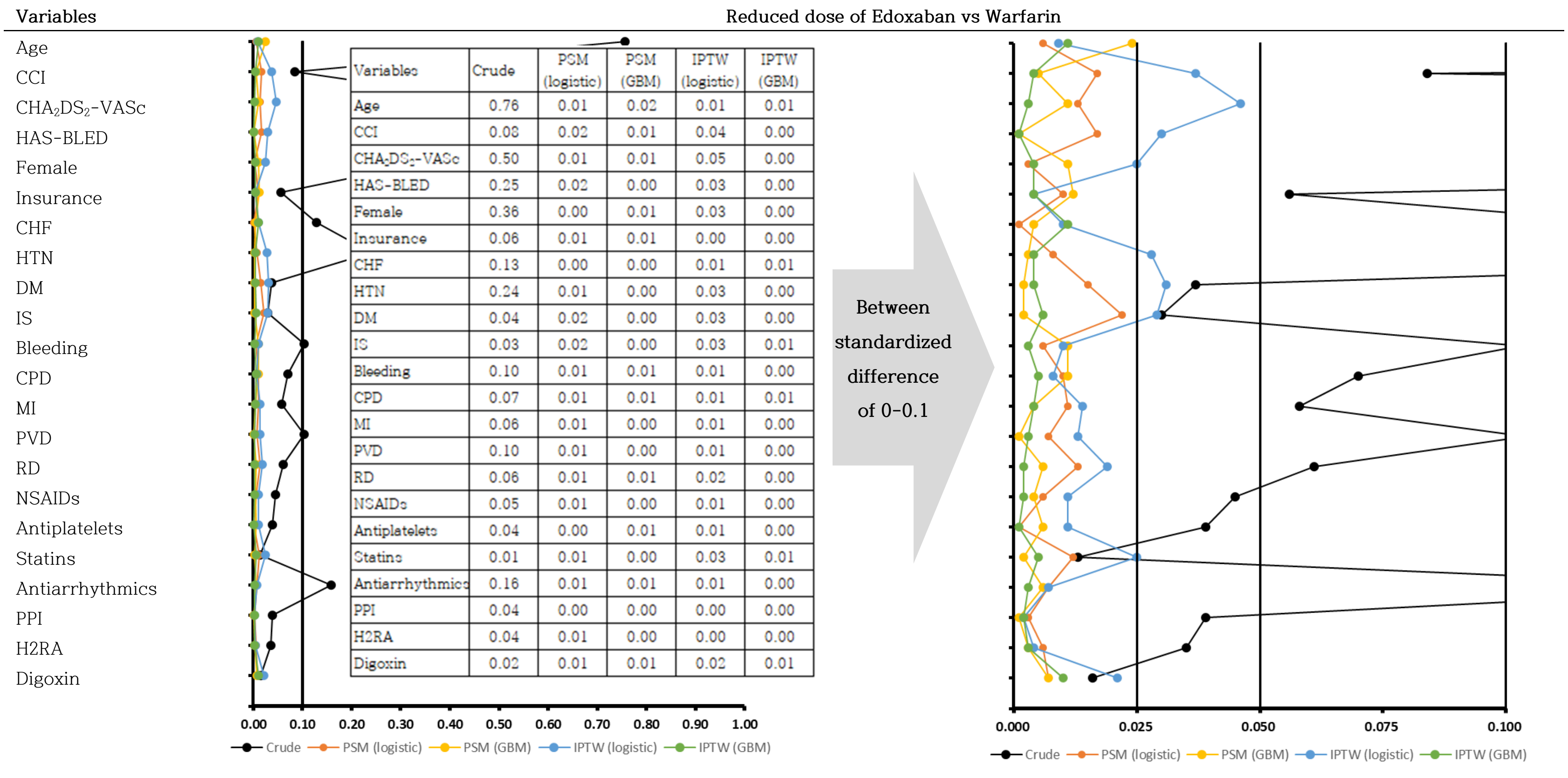

Variables	Crude	PSM (logistic)	PSM (GBM)	IPTW (logistic)	IPTW (GBM)
Age	0.76	0.01	0.02	0.01	0.01
CCI	0.08	0.02	0.01	0.04	0.00
CHA_2DS_2-VASc	0.50	0.01	0.01	0.05	0.00
HAS-BLED	0.25	0.02	0.00	0.03	0.00
Female	0.36	0.00	0.01	0.03	0.00
Insurance	0.06	0.01	0.01	0.00	0.00
CHF	0.13	0.00	0.00	0.01	0.01
HTN	0.24	0.01	0.00	0.03	0.00
DM	0.04	0.02	0.00	0.03	0.00
IS	0.03	0.02	0.00	0.03	0.01
Bleeding	0.10	0.01	0.01	0.01	0.00
CPD	0.07	0.01	0.01	0.01	0.01
MI	0.06	0.01	0.00	0.01	0.00
PVD	0.10	0.01	0.00	0.01	0.00
RD	0.06	0.01	0.01	0.02	0.00
NSAIDs	0.05	0.01	0.00	0.01	0.00
Antiplatelets	0.04	0.00	0.01	0.01	0.00
Statins	0.01	0.01	0.00	0.03	0.01
Antiarrhythmics	0.16	0.01	0.01	0.01	0.00
PPI	0.04	0.00	0.00	0.00	0.00
H2RA	0.04	0.01	0.00	0.00	0.00
Digoxin	0.02	0.01	0.01	0.02	0.01

CCI, Charson Comorbidity Index; CHA2DS2-VASc, congestive heart failure, hypertension, age ≥75 years, diabetes mellitus, stroke, vascular disease, age 65–74 years, and sex; CHF, congestive heart failure; CPD, chronic pulmonary disease; DM, diabetes mellitus; HAS-BLED, hypertension, abnormal renal and liver function, stroke, bleeding, labile international normalized ratio, elderly, drugs, or alcohol; H2RA, H2-receptor antagonist; HTN, hypertension; IS, ischemic stroke; IPTW, inverse probability of treatment weighting; MI, myocardial infarction; PPI, proton pump inhibitor; PSM, propensity score matching; PVD, peripheral vascular disease; RD, renal disease. Conventional approaches are PSM (logistic) and IPTW (logistic) and machine learning approaches are PSM (GBM) and IPTW (GBM).

Supplementary Figure S6. Absolute standardized differences in comparison 6

Variables	Standard dose of Rivaroxaban vs Warfarin

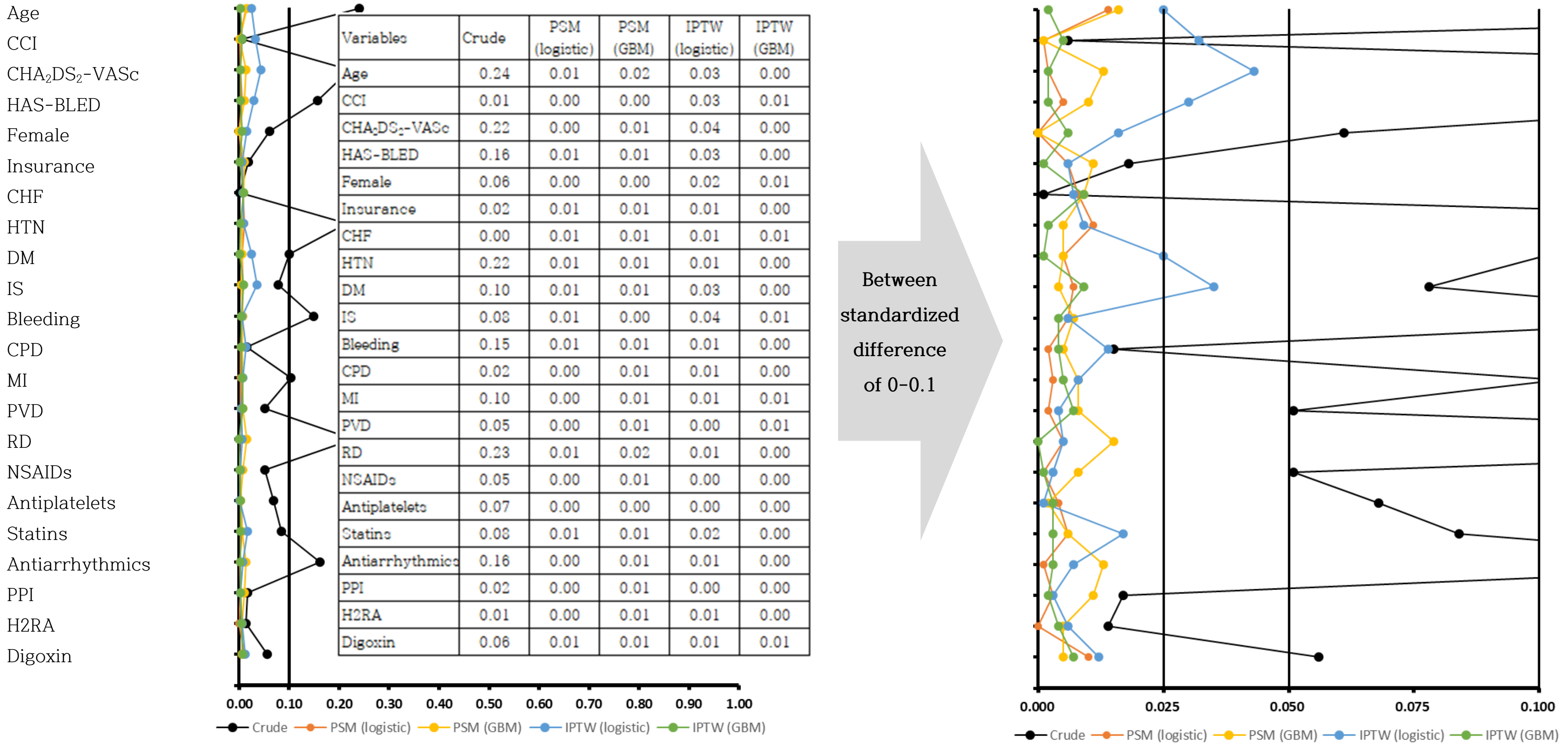

Variables	Crude	PSM (logistic)	PSM (GBM)	IPTW (logistic)	IPTW (GBM)
Age	0.24	0.01	0.02	0.03	0.00
CCI	0.01	0.00	0.00	0.03	0.01
CHA_2DS_2-VASc	0.22	0.00	0.01	0.04	0.00
HAS-BLED	0.16	0.01	0.01	0.03	0.00
Female	0.06	0.00	0.00	0.02	0.01
Insurance	0.02	0.01	0.01	0.01	0.00
CHF	0.00	0.01	0.01	0.01	0.01
HTN	0.22	0.01	0.01	0.01	0.00
DM	0.10	0.01	0.01	0.03	0.00
IS	0.08	0.01	0.00	0.04	0.01
Bleeding	0.15	0.01	0.01	0.01	0.00
CPD	0.02	0.00	0.01	0.01	0.00
MI	0.10	0.00	0.01	0.01	0.01
PVD	0.05	0.00	0.01	0.00	0.01
RD	0.23	0.01	0.02	0.01	0.00
NSAIDs	0.05	0.00	0.01	0.00	0.00
Antiplatelets	0.07	0.00	0.00	0.00	0.00
Statins	0.08	0.01	0.01	0.02	0.00
Antiarrhythmics	0.16	0.00	0.01	0.01	0.00
PPI	0.02	0.00	0.01	0.00	0.00
H2RA	0.01	0.00	0.01	0.01	0.00
Digoxin	0.06	0.01	0.01	0.01	0.01

CCI, Charson Comorbidity Index; CHA2DS2-VASc, congestive heart failure, hypertension, age ≥75 years, diabetes mellitus, stroke, vascular disease, age 65–74 years, and sex; CHF, congestive heart failure; CPD, chronic pulmonary disease; DM, diabetes mellitus; HAS-BLED, hypertension, abnormal renal and liver function, stroke, bleeding, labile international normalized ratio, elderly, drugs, or alcohol; H2RA, H2-receptor antagonist; HTN, hypertension; IS, ischemic stroke; IPTW, inverse probability of treatment weighting; MI, myocardial infarction; PPI, proton pump inhibitor; PSM, propensity score matching; PVD, peripheral vascular disease; RD, renal disease. Conventional approaches are PSM (logistic) and IPTW (logistic) and machine learning approaches are PSM (GBM) and IPTW (GBM).

Supplementary Figure S7. Absolute standardized differences in comparison 7

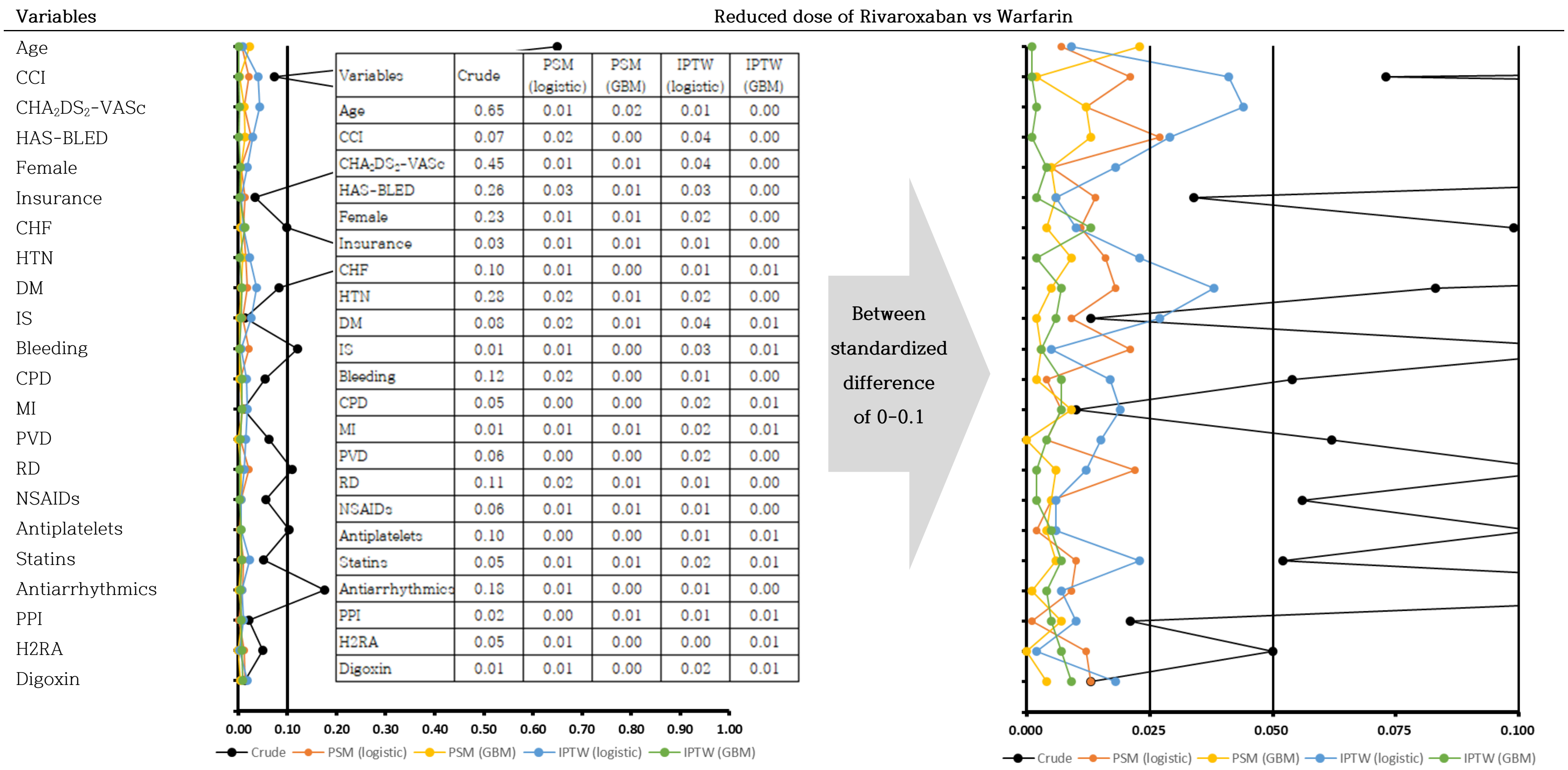

Variables	Crude	PSM (logistic)	PSM (GBM)	IPTW (logistic)	IPTW (GBM)
Age	0.65	0.01	0.02	0.01	0.00
CCI	0.07	0.02	0.00	0.04	0.00
CHA_2DS_2-VASc	0.45	0.01	0.01	0.04	0.00
HAS-BLED	0.26	0.03	0.01	0.03	0.00
Female	0.23	0.01	0.01	0.02	0.00
Insurance	0.03	0.01	0.01	0.01	0.00
CHF	0.10	0.01	0.00	0.01	0.01
HTN	0.28	0.02	0.01	0.02	0.00
DM	0.08	0.02	0.01	0.04	0.01
IS	0.01	0.01	0.00	0.03	0.01
Bleeding	0.12	0.02	0.00	0.01	0.00
CPD	0.05	0.00	0.00	0.02	0.01
MI	0.01	0.01	0.01	0.02	0.01
PVD	0.06	0.00	0.00	0.02	0.00
RD	0.11	0.02	0.01	0.01	0.00
NSAIDs	0.06	0.01	0.01	0.01	0.00
Antiplatelets	0.10	0.00	0.00	0.01	0.01
Statins	0.05	0.01	0.01	0.02	0.01
Antiarrhythmics	0.18	0.01	0.00	0.01	0.00
PPI	0.02	0.00	0.01	0.01	0.01
H2RA	0.05	0.01	0.00	0.00	0.01
Digoxin	0.01	0.01	0.00	0.02	0.01

CCI, Charson Comorbidity Index; CHA2DS2-VASc, congestive heart failure, hypertension, age ≥75 years, diabetes mellitus, stroke, vascular disease, age 65–74 years, and sex; CHF, congestive heart failure; CPD, chronic pulmonary disease; DM, diabetes mellitus; HAS-BLED, hypertension, abnormal renal and liver function, stroke, bleeding, labile international normalized ratio, elderly, drugs, or alcohol; H2RA, H2-receptor antagonist; HTN, hypertension; IS, ischemic stroke; IPTW, inverse probability of treatment weighting; MI, myocardial infarction; PPI, proton pump inhibitor; PSM, propensity score matching; PVD, peripheral vascular disease; RD, renal disease. Conventional approaches are PSM (logistic) and IPTW (logistic) and machine learning approaches are PSM (GBM) and IPTW (GBM).

Supplementary Figure S8. Absolute standardized differences in comparison 8

Methods	Groups	Age (years) at the index date
Crude	S.Apixaban	
	Warfarin	
PSM (logistic)	S.Apixaban	
	Warfarin	
PSM (GBM)	S.Apixaban	
	Warfarin	
IPTW (logistic)	S.Apixaban	
	Warfarin	
IPTW (GBM)	S.Apixaban	
	Warfarin	

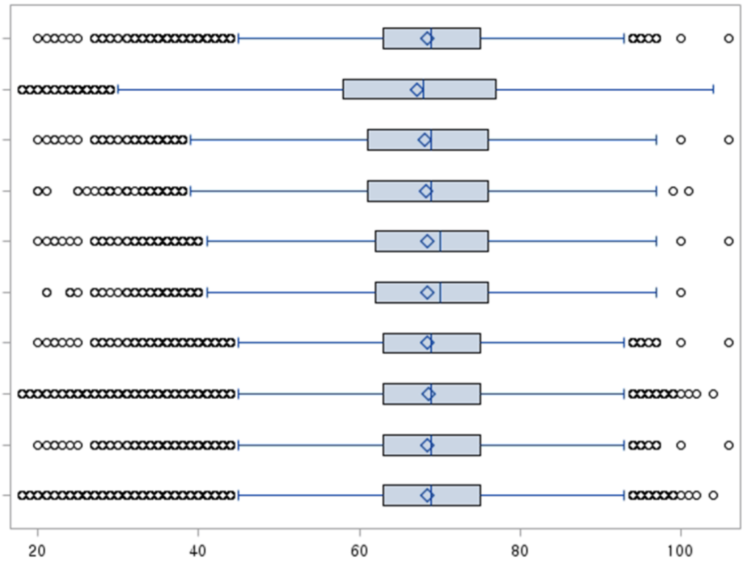

		CHA_2DS_2-VASc score
Crude	S.Apixaban	
	Warfarin	
PSM (logistic)	S.Apixaban	
	Warfarin	
PSM (GBM)	S.Apixaban	
	Warfarin	
IPTW (logistic)	S.Apixaban	
	Warfarin	
IPTW (GBM)	S.Apixaban	
	Warfarin	

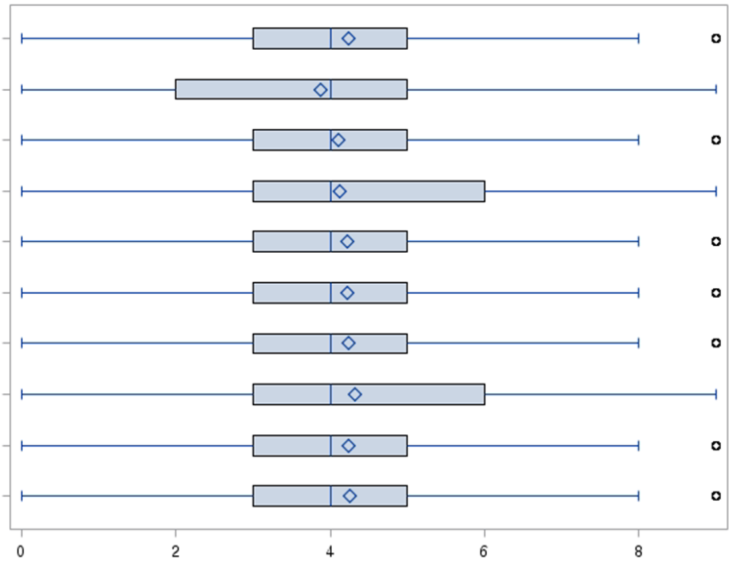

S, standard dose of.
* Conventional approach: PSM (logistic) and IPTW (logistic).
* Machine learning approach: PSM (GBM) and IPTW (GBM).

Supplementary Figure S9. Side-by-side boxplot in comparison 1

Methods	Groups	Age (years) at the index date
Crude	R.Apixaban	
	Warfarin	
PSM (logistic)	R.Apixaban	
	Warfarin	
PSM (GBM)	R.Apixaban	
	Warfarin	
IPTW (logistic)	R.Apixaban	
	Warfarin	
IPTW (GBM)	R.Apixaban	
	Warfarin	

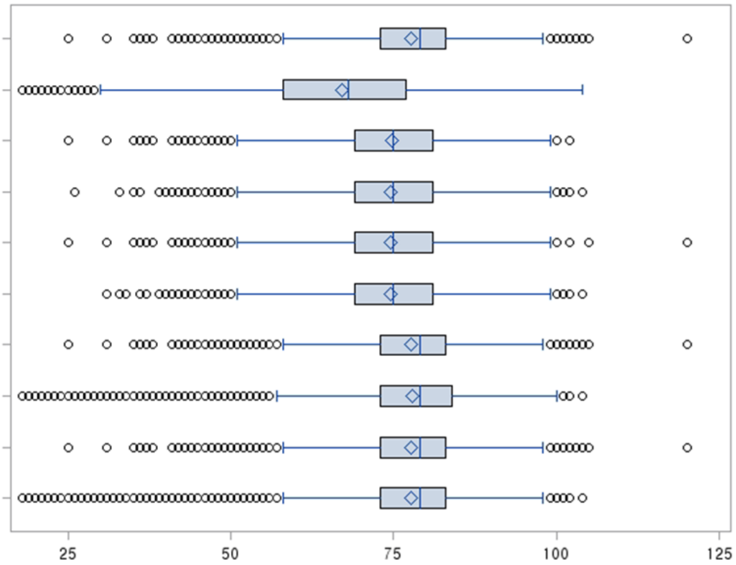

		CHA_2DS_2-VASc score
Crude	R.Apixaban	
	Warfarin	
PSM (logistic)	R.Apixaban	
	Warfarin	
PSM (GBM)	R.Apixaban	
	Warfarin	
IPTW (logistic)	R.Apixaban	
	Warfarin	
IPTW (GBM)	R.Apixaban	
	Warfarin	

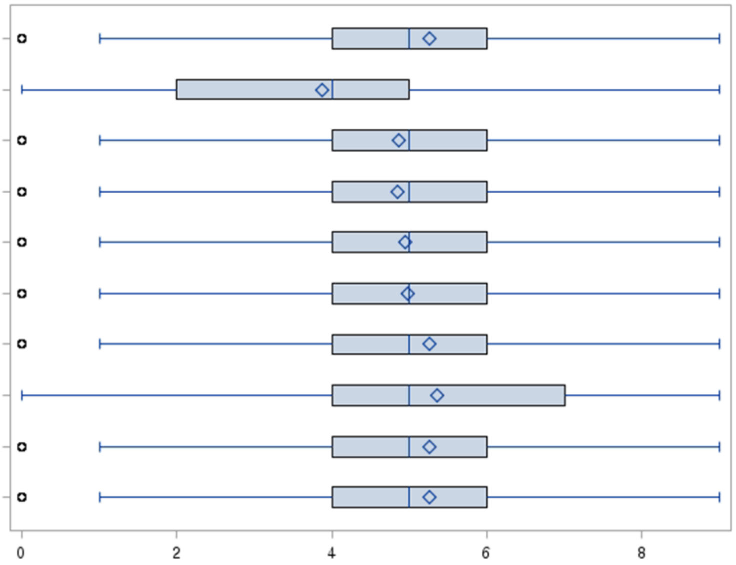

R, reduced dose of.
* Conventional approach: PSM (logistic) and IPTW (logistic).
* Machine learning approach: PSM (GBM) and IPTW (GBM).

Supplementary Figure S10. Side-by-side boxplot in comparison 2

Methods	Groups	Age (years) at the index date
Crude	S.Dabigatran	
	Warfarin	
PSM (logistic)	S.Dabigatran	
	Warfarin	
PSM (GBM)	S.Dabigatran	
	Warfarin	
IPTW (logistic)	S.Dabigatran	
	Warfarin	
IPTW (GBM)	S.Dabigatran	
	Warfarin	

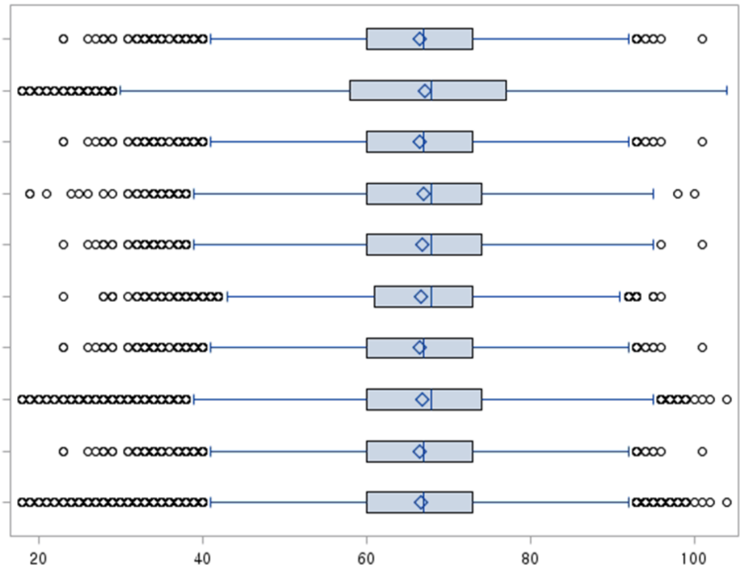

		CHA_2DS_2-VASc score
Crude	S.Dabigatran	
	Warfarin	
PSM (logistic)	S.Dabigatran	
	Warfarin	
PSM (GBM)	S.Dabigatran	
	Warfarin	
IPTW (logistic)	S.Dabigatran	
	Warfarin	
IPTW (GBM)	S.Dabigatran	
	Warfarin	

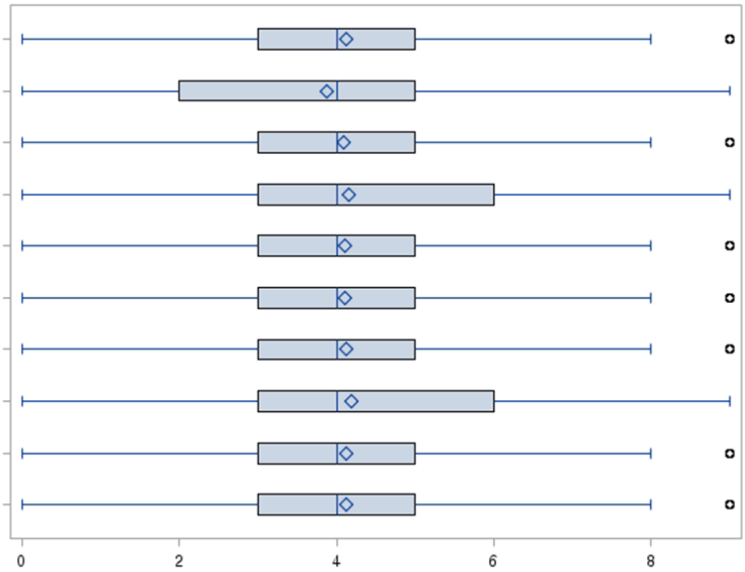

S, standard dose of.
* Conventional approach: PSM (logistic) and IPTW (logistic).
* Machine learning approach: PSM (GBM) and IPTW (GBM).

Supplementary Figure S11. Side-by-side boxplot in comparison 3

Methods	Groups	Age (years) at the index date
Crude	R.Dabigatran	
	Warfarin	
PSM (logistic)	R.Dabigatran	
	Warfarin	
PSM (GBM)	R.Dabigatran	
	Warfarin	
IPTW (logistic)	R.Dabigatran	
	Warfarin	
IPTW (GBM)	R.Dabigatran	
	Warfarin	

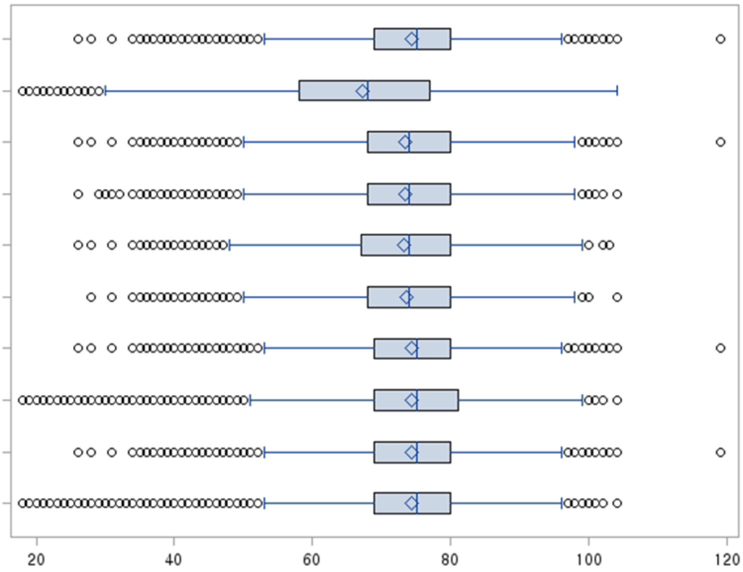

Methods	Groups	CHA_2DS_2-VASc score
Crude	R.Dabigatran	
	Warfarin	
PSM (logistic)	R.Dabigatran	
	Warfarin	
PSM (GBM)	R.Dabigatran	
	Warfarin	
IPTW (logistic)	R.Dabigatran	
	Warfarin	
IPTW (GBM)	R.Dabigatran	
	Warfarin	

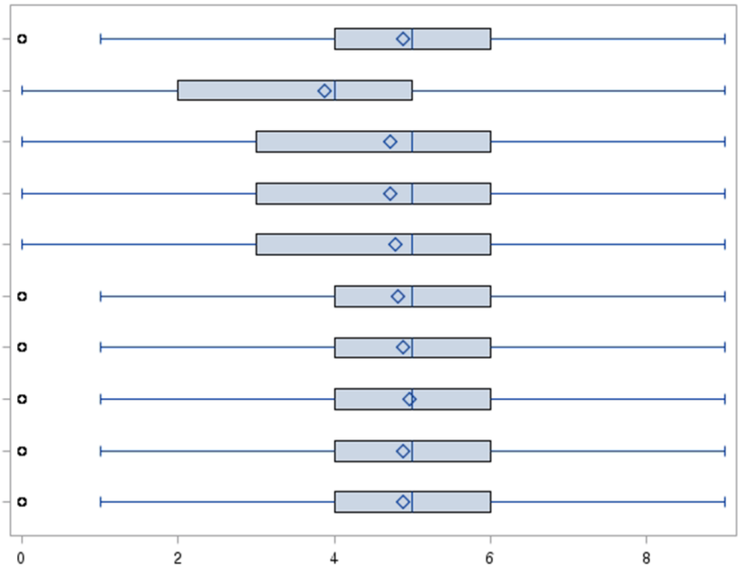

R, reduced dose of.
* Conventional approach: PSM (logistic) and IPTW (logistic).
* Machine learning approach: PSM (GBM) and IPTW (GBM).

Supplementary Figure S12. Side-by-side boxplot in comparison 4

Methods	Groups	Age (years) at the index date
Crude	S.Edoxaban	
	Warfarin	
PSM (logistic)	S.Edoxaban	
	Warfarin	
PSM (GBM)	S.Edoxaban	
	Warfarin	
IPTW (logistic)	S.Edoxaban	
	Warfarin	
IPTW (GBM)	S.Edoxaban	
	Warfarin	

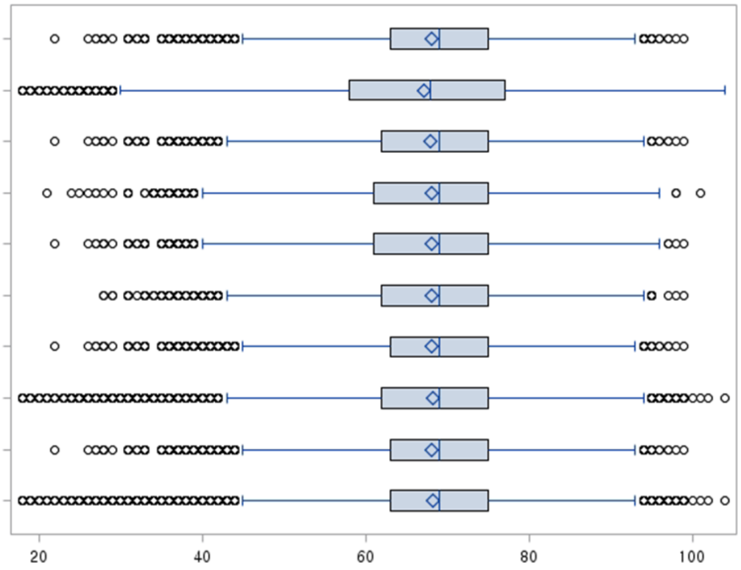

Methods	Groups	CHA_2DS_2-VASc score
Crude	S.Edoxaban	
	Warfarin	
PSM (logistic)	S.Edoxaban	
	Warfarin	
PSM (GBM)	S.Edoxaban	
	Warfarin	
IPTW (logistic)	S.Edoxaban	
	Warfarin	
IPTW (GBM)	S.Edoxaban	
	Warfarin	

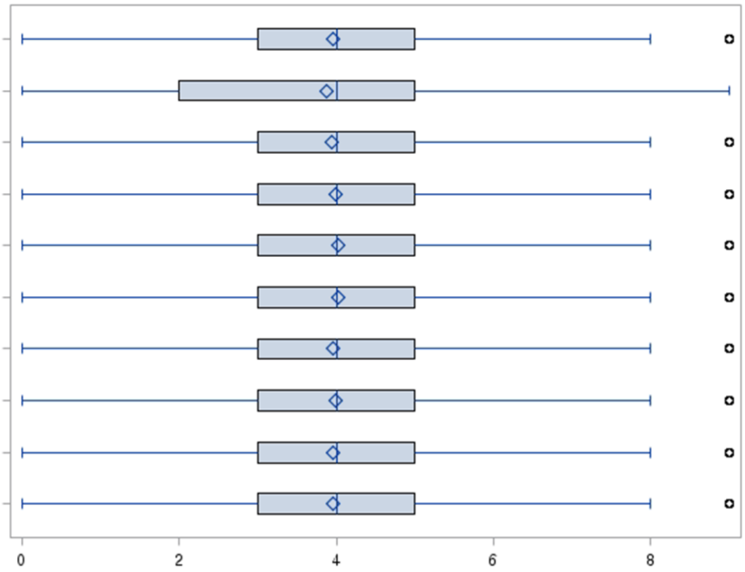

S, standard dose of.
* Conventional approach: PSM (logistic) and IPTW (logistic).
* Machine learning approach: PSM (GBM) and IPTW (GBM).

Supplementary Figure S13. Side-by-side boxplot in comparison 5

Methods	Groups	Age (years) at the index date
Crude	R.Edoxaban	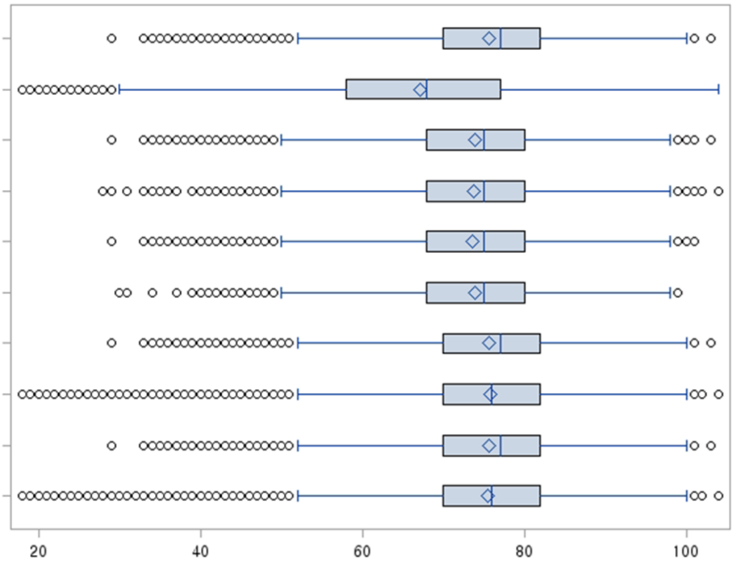
	Warfarin	
PSM (logistic)	R.Edoxaban	
	Warfarin	
PSM (GBM)	R.Edoxaban	
	Warfarin	
IPTW (logistic)	R.Edoxaban	
	Warfarin	
IPTW (GBM)	R.Edoxaban	
	Warfarin	

		CHA_2DS_2-VASc score
Crude	R.Edoxaban	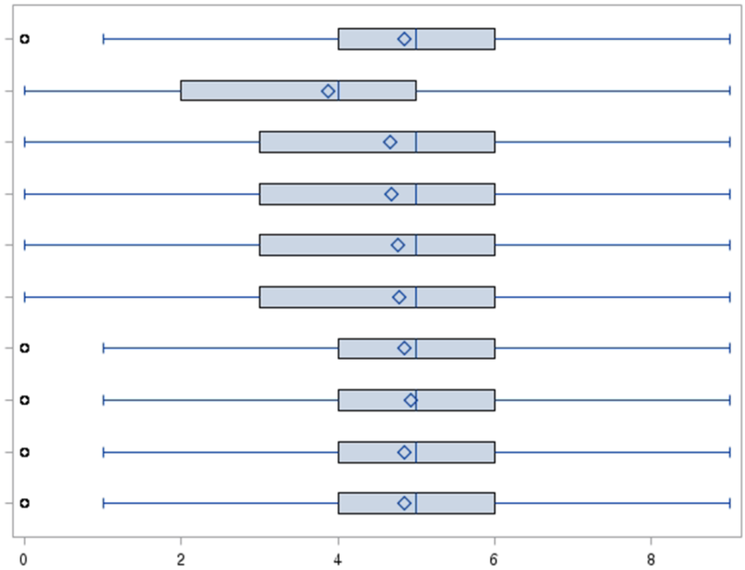
	Warfarin	
PSM (logistic)	R.Edoxaban	
	Warfarin	
PSM (GBM)	R.Edoxaban	
	Warfarin	
IPTW (logistic)	R.Edoxaban	
	Warfarin	
IPTW (GBM)	R.Edoxaban	
	Warfarin	

R. reduced dose of.
* Conventional approach: PSM (logistic) and IPTW (logistic).
* Machine learning approach: PSM (GBM) and IPTW (GBM).

Supplementary Figure S14. Side-by-side boxplot in comparison 6

Methods	Groups
Crude	S.Rivaroxaban
	Warfarin
PSM (logistic)	S.Rivaroxaban
	Warfarin
PSM (GBM)	S.Rivaroxaban
	Warfarin
IPTW (logistic)	S.Rivaroxaban
	Warfarin
IPTW (GBM)	S.Rivaroxaban
	Warfarin

Age (years) at the index date

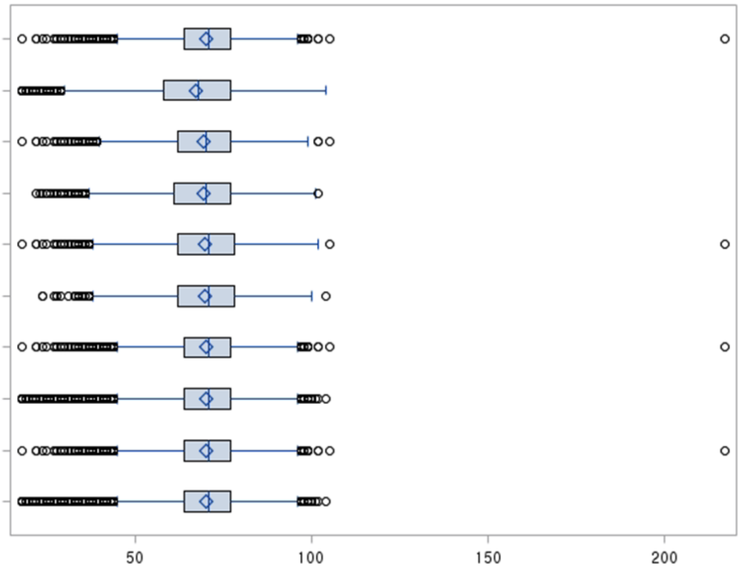

CHA_2DS_2-VASc score

Methods	Groups
Crude	S.Rivaroxaban
	Warfarin
PSM (logistic)	S.Rivaroxaban
	Warfarin
PSM (GBM)	S.Rivaroxaban
	Warfarin
IPTW (logistic)	S.Rivaroxaban
	Warfarin
IPTW (GBM)	S.Rivaroxaban
	Warfarin

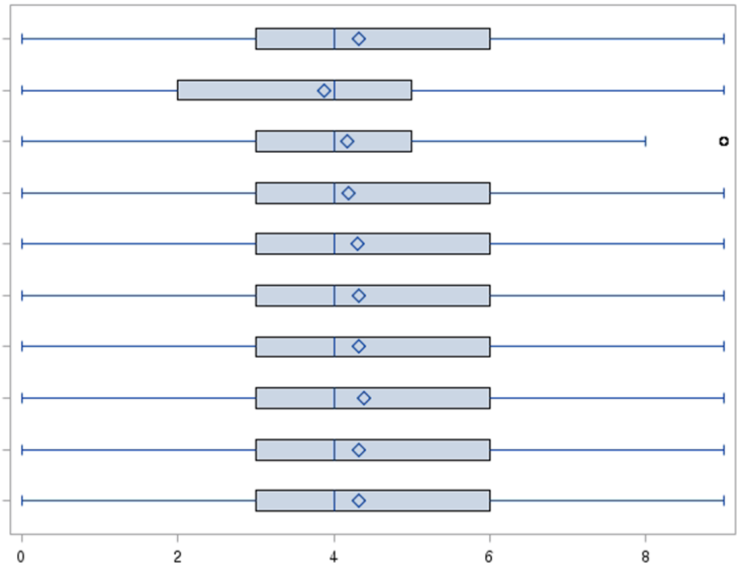

S, standard dose of.
* Conventional approach: PSM (logistic) and IPTW (logistic).
* Machine learning approach: PSM (GBM) and IPTW (GBM).

Supplementary Figure S15. Side-by-side boxplot in comparison 7

Methods	Groups	Age (years) at the index date
Crude	R.Rivaroxaban	
	Warfarin	
PSM (logistic)	R.Rivaroxaban	
	Warfarin	
PSM (GBM)	R.Rivaroxaban	
	Warfarin	
IPTW (logistic)	R.Rivaroxaban	
	Warfarin	
IPTW (GBM)	R.Rivaroxaban	
	Warfarin	

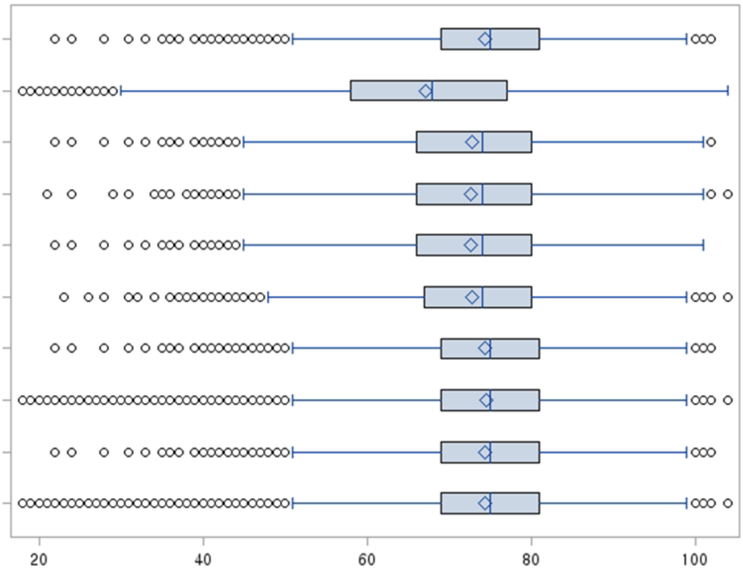

		CHA_2DS_2-VASc score
Crude	R.Rivaroxaban	
	Warfarin	
PSM (logistic)	R.Rivaroxaban	
	Warfarin	
PSM (GBM)	R.Rivaroxaban	
	Warfarin	
IPTW (logistic)	R.Rivaroxaban	
	Warfarin	
IPTW (GBM)	R.Rivaroxaban	
	Warfarin	

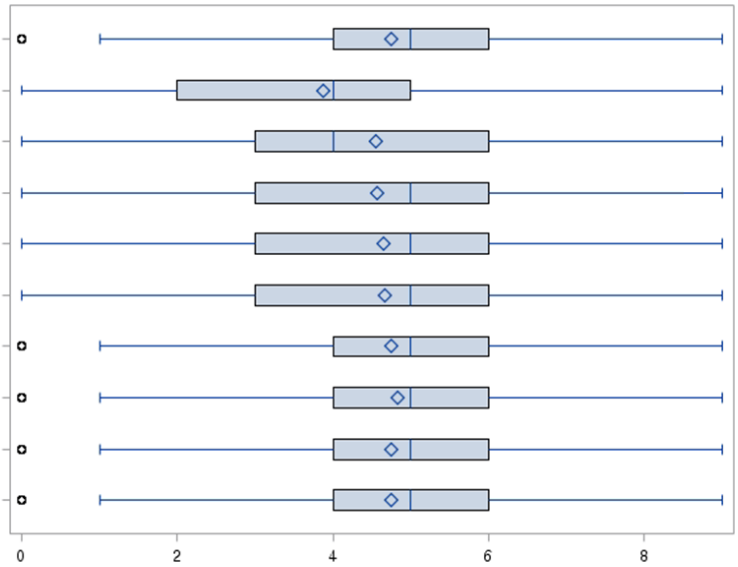

R, reduced dose of.
* Conventional approach: PSM (logistic) and IPTW (logistic).
* Machine learning approach: PSM (GBM) and IPTW (GBM).

Supplementary Figure S16. Side-by-side boxplot in comparison 8
